# Feeding of fish oil and medium-chain triglycerides to canines impacts circulating structural and energetic lipids, endocannabinoids, and non-lipid metabolite profiles

**DOI:** 10.3389/fvets.2023.1168703

**Published:** 2023-08-24

**Authors:** Matthew I. Jackson, Dennis E. Jewell

**Affiliations:** ^1^Pet Nutrition Center, Hill's Pet Nutrition, Inc., Topeka, KS, United States; ^2^Department of Grain Science and Industry, Kansas State University, Manhattan, KS, United States

**Keywords:** canine, fish oil, medium-chain triglycerides, metabolome, lipidome, endocannabinoid

## Abstract

**Introduction:**

The effect of medium-chain fatty acid-containing triglycerides (MCT), long-chain polyunsaturated fatty acid-containing triglycerides from fish oil (FO), and their combination (FO+MCT) on the serum metabolome of dogs (*Canis familiaris*) was evaluated.

**Methods:**

Dogs (*N* = 64) were randomized to either a control food, one with 7% MCT, one with FO (0.18% eicosapentaenoate and 1.3% docosahexaenoate), or one with FO+MCT for 28 days following a 14-day washout period on the control food. Serum metabolites were analyzed via chromatography followed by mass spectrometry.

**Results:**

Additive effects of serum metabolites were observed for a number of metabolite classes, including fatty acids, phospholipids, acylated amines including endocannabinoids, alpha-oxidized fatty acids, and methyl donors. Some effects of the addition of FO+MCT were different when the oils were combined compared with when each oil was fed separately, namely for acylcarnitines, omega-oxidized dicarboxylic acids, and amino acids. Several potentially beneficial effects on health were observed, including decreased circulating triglycerides and total cholesterol with the addition of FO (with or without MCT) and decreases in N-acyl taurines with the addition of MCT, FO, or FO+MCT.

**Discussion:**

Overall, the results of this study provide a phenotypic characterization of the serum lipidomic response to dietary supplementation of long-chain n3-polyunsaturated and medium-chain saturated fats in canines.

## 1. Introduction

The impact of the gut microbiome on host development, health, and metabolism has been well-studied in the last few decades, with dietary factors affecting the composition and function of the microbiome in both companion animals (including dogs) and humans (1–3). Several of these studies have linked the composition of the gut microbiome with circulating lipids in humans (3, 4). Medium-chain triglycerides (MCTs) and long-chain polyunsaturated triglycerides (LCPUTs) are dietary fatty acids with demonstrated positive health effects. While each can provide dietary energy, they can also affect physiology.

Prior study has shown that MCT can cause changes in the gut microbiome in a mouse model and in pigs (5, 6). In addition to the effects on the microbiome, MCT confers positive effects on the intestine. MCT supplementation appeared to protect rats from endotoxemia, preventing mortality, and injury to the gut and liver following lipopolysaccharide administration (7) and improving chemically induced colitis in rats (8). Supplementation with capric acid resulted in positive changes in the structure of the ileal mucosal epithelium (9) and protected against induced intestinal oxidative stress, inflammation, and barrier function, both studied in pigs (10). These effects may have implications on circulatory metabolites of microbial origin.

The n3 long-chain polyunsaturated fatty acids [LCPUFA(n3)] have frequently been tested in canine foods and have shown to be effective in changing many relevant biological outcomes. Both LCPUFA(n6) (11) and LCPUFA(n3) (12) are essential fatty acids in dogs. Fish oil (FO), which is high in LCPUT, can affect the composition of the gut microbiome as seen in studies on obesity (13, 14) and brain aging (15) in mice. Foods high in docosahexaenoic acid (DHA; C22:6n3) were shown to improve cognitive learning, immunological, and retinal function in puppies (16). FO with an eicosapentaenoate (EPA; C20:5n3)/DHA ratio of approximately 1.5 was effective at reducing urinary 11-dehydro thromboxane B_2_ concentration in dogs, supporting its effectiveness in reducing inflammation (17). EPA and DHA have been shown to aid in the management of osteoarthritis (18–20), including reducing the needed medications in managing this disease (21). EPA and DHA also confer anti-inflammatory effects (22) and lead to modifications in the gut microbiome in both humans and animal models (13, 23). Consumption of FO by dogs enriches the composition of circulating complex lipids with DHA (24). As with MCT, LCPUFA(n3) appear to exert protective effects on gut epithelial barrier function in an *in vitro* model (25). The LCPUFA(n6) linoleate (18:2n6) is considered essential (26), and its levels decrease in the skin of dogs with ichthyosis (27). The ratio of n6/n3 LCPUFA may be a determinant of the degree to which these LCPUFAs are beneficial (28). In some contexts, LCPUFA(n6) may be detrimental to gut health, as shown through the modification of the gut microbiome in a mouse model (29). Whereas FO has been used therapeutically to manage canine disease related to immune status and inflammation, MCTs have been employed in companion animal dogs as a therapeutic intervention to aid in the management of seizures (30, 31) and cognitive decline (32). MCT combined with FO appears to decrease inflammation (33, 34) in mice, may modulate risk factors of cardiovascular disease (35) in a rat model, and may abate age-related changes in circulating concentrations of fatty acids and carnitine metabolites in dogs (36). The combination of MCT and FO has also been used to manage myxomatous mitral valve heart disease in dogs (37). However, in that study, FO was predominantly composed of EPA with relatively little DHA. A previous study in cats tested the effects of foods including MCT, FO, or both on the plasma metabolome and found a combined effect on several lipid classes, including those derived from gut microbial metabolism (38).

Canine physiological states share commonality with human counterparts; for example, there are parallels between the physiology of human and canine aging (39) and obesity (40, 41). As well, dogs are a model for human gestational diabetes (42), insulin-dependent diabetes mellitus (type 1) (43), and glucocorticoid therapy (44). Dogs are also prone to endocrine diseases that afflict humans, including non-insulin-dependent diabetes mellitus (type 2) (40), Cushing disease (45), and hypothyroidism (46). Dogs in these life stages, disease states, and conditions present with altered lipid profiles and dyslipidemia similar to humans. Aging and obesity elevate triglycerides in both humans (47, 48) and dogs (49, 50). Progression and outcome of human type 2 diabetes are influenced by diet and are associated with hypercholesterolemia (51); canine type 2 diabetes is also impacted by diet type (52), and experimental canine diabetes manifests hypercholesterolemia (43), although the organ-specific contributions to cholesterol accretion differ between species. Excess levels of glucocorticoids promote dyslipidemia (53) and changes in the circulating complex lipids (en toto, the “lipidome”) that accompany acute and chronic glucocorticoid or adrenocorticotropin hormone provision have recently been described in dogs (54). Changes in the circulating canine lipidome accompanying Cushing disease and hypothyroidism have also recently been investigated in dogs (55). In contrast with humans, dogs are normally resistant to atherosclerosis even in the presence of obesity and dyslipidemia (56). Intriguingly, dogs with atherosclerosis were more likely to have concurrent diabetes or hypothyroidism (57), similar to observations for humans with hypothyroidism (58) and diabetes (59).

Representative lipidomes are similar between dogs and humans in ocular (60) and synovial (61) matrices. Despite these commonalities, variations in the circulating lipidome can differentiate breed types (62). Examinations of the lipidome have shown utility in studying genetic (63) and pharmacologic (54) canine models of disease. The canine lipidome has also been assessed in naturally occurring inflammatory diseases, including atopic dermatitis (64), chronic gastroenteritis (65), and the aforementioned endocrine diseases (55). The impact of dietary LCPUFA(n3), including DHA and EPA, on classes of metabolites within the canine lipidome has been reported (66), including a study that monitored the canine lipidome during a dietary feeding study with increased n6- and n3-PUFA in dogs with enteritis (67). In the present study, healthy dogs were chosen as test subjects as they have been previously assessed for response to intake of dietary lipids (36, 66, 68, 69). While these studies showed that food can modify some metabolomic parameters, the global lipidome response was not reported (36, 68, 69), control and test foods were not matched for ingredients and nutrition (66), and/or the effects of MCT with FO both alone and in combination were not compared (36, 66, 68, 69).

Here, dog food was supplemented with FO, MCT, or both as part of a complete maintenance food balanced for total dietary fat, protein, and carbohydrates in order to determine the impact of these dietary oils on the canine serum lipidome. This is the first study to report levels of several classes of lipid metabolites examined, including non-esterified fatty acids as well as their glycerides, acylcarnitines, amide endocannabinoids, and structural phospholipids. The fatty acid oxidation products canonically produced by the mitochondria (beta-hydroxy), endoplasmic reticulum (alpha-hydroxy), peroxisome (dioate), and membrane (e.g., hydroxyeicosatetraenoate [HETE]) are also reported. Changes in circulating proxies of central metabolites including those of the tricarboxylic acid cycle (TCA) and amino acids were analyzed to gain insight into the degree to which the dietary fats were impacting associated energetic pathways. Circulating putrefactive postbiotics and their host-conjugated sulfates were also evaluated, particularly as our previous study showed that co-consumption of FO+MCT in domestic cats results in a decrease in circulating postbiotics of the indole and phenol classes (38).

## 2. Materials and methods

### 2.1. Ethics statement

The Institutional Animal Care and Use Committee, Hill's Pet Nutrition, Topeka, KS, USA (Protocol Number: FP578.1.2.0-A-C-D-ADH) reviewed and approved the study protocol. The study also complied with the National Institutes of Health guide for the care and use of laboratory animals and the guides from the US National Research Council and the US Public Health Service (70). Healthy dogs were included in the study, defined as those without chronic systemic disease based on physical examination, complete blood count, serum biochemical analyses, urinalysis, and fecal examination for parasites. No invasive procedures were used in this study.

### 2.2. Food formulation and production

The four dry extruded test foods used in the study were composed of the same base formula primarily of poultry byproduct meal, wet chicken meat, pork fat, barley, corn gluten meal, whole corn, wheat, and sorghum, as well as liver hydrolysate, fiber, vitamins, and minerals (Supplementary Table 1). MCT, FO, or both replaced pork fat levels in the test foods. As previously (38), CAPTEX-355 (ViaChem Inc, Plano, TX, USA) was the source of MCT, which is enriched for caprylate (C8:0) over caprate (C10:0), and caproate (C6:0) with negligible amounts of laurate (C12:0) and myristate (C14:0). Third-party testing (Eurofins Nutrition Analysis Center, Des Moines, IA, USA) showed the composition to be 51.4% caprylate (C8:0), 39.1% caprate (C10:0), < 0.1% laurate (C12:0), and < 0.01% each of all other fatty acids. Caproate (C6:0) was not reported but is projected to be approximately 8%. MEG-3^TM^ 0355TG Oil (DSM Inc., Parsippany, NJ, USA) was used as the FO source of LCPUT(n3) as it is enriched for DHA (C22:6n3; 36.5%) over EPA (C20:5n3; 5%). All four foods used in this study met the canine maintenance nutrition requirements of the Association of American Feed Control Officials and National Research Council.

The test oils, MCT and FO, were added to the foods on a dry matter basis to be 7 and 2.85%, respectively. The MCT level was chosen to provide >20% of total dietary fat as MCT, similar to levels in prior publications that tested dietary MCT supplementation in dogs (71, 72). FO was fed at a dietary inclusion level previously found to be safe, as demonstrated in canine feeding trials that employed approximately 100 mg/kg body weight (73, 74). Based on certified analysis of ingredients and formulation levels, the MCT-containing foods had 3.7% caprylate (C8:0) and 4.3% caprate (C10:0), while the FO-containing foods had 0.18% EPA (C20:5n3) and 1.3% DHA (C22:6n3), all on a dry matter basis (Supplementary Table 2).

### 2.3. Study design and measurements

Animal care research technicians and sample analysts were blinded to the foods provided and to the group identity of dogs for purposes of sample collection and analysis. Dogs were beagles or mixed breeds (Supplementary Table 3), owned by the funders of this research, and acquired from on-site husbandry or licensed breeders. The sample size (*N* = 64) was based on effect sizes from a previous study (36). It was designed for 80% power to detect a 20% difference between groups for selected lipids while allowing for a potential dropout rate of 5% and a need for correction for multiple between-group testing. The study had a 2 × 2 factorial design. During the washout period, all dogs were fed the control (CON) food for 14 days. Dogs were then randomized into one of four foods (*n* = 16 each; CON, MCT, FO, FO+MCT) for 28 days by distributing the dogs into groups based on breed, sex, weight, and age; there were no significant differences in these parameters across groups (*P* > 0.7 for all). All pets had the opportunity for exercise and interaction together in large groups (~20 dogs) but were pair-housed for sleeping arrangements. Dogs remained in their preferred housing arrangement during the trial as previously determined by the colony veterinarian's assessment of temperament and social interactions. Dogs were fed daily at electronic feeders where each pet (through a radio frequency identification chip reader) was individually given access to food for 1 h of a controlled amount. These electronic feeders recorded food intake (g/day) for each dog. Dogs were fed to maintain body weights from the start of the study, which was a mean ± SD metabolizable energy of 1.69 ± 0.40 × 70 kcal × (kg body weight [BW])^0.75^; water was available *ad libitum*.

Serum was collected prior to consumption of test foods at the end of the washout period to serve as a D0 baseline (D0) and again at day 28 (D28) at the end of the feeding period. Dogs were fasted for 23 h prior to serum collection, in which the total amount of blood drawn was 14 mL. Clinical blood chemistry was carried out on a COBAS c501 module (Roche Diagnostics Corporation, Indianapolis, IN, USA), and analysis of serum metabolomics was performed by Metabolon (Morrisville, NC, USA) as in previous studies (38, 75, 76).

### 2.4. Statistical analysis

As described previously (38), metabolite values were natural log (LN)-transformed, and LN(D0) baseline values were subtracted from LN(D28) end-of-feeding period values to create the difference of logs [LN(D28)–LN(D0)]. This difference of the log values is mathematically equivalent to, and is presented here, as the LN fold change [LN(D28)–LN(D0)] (77). This data normalization approach also conveniently results in positive values when the D28 value for a metabolite is greater than at the D0 baseline, while negative values on the y-axis indicate that a given metabolite has decreased from the D0 baseline. As each dog had values for both D0 baseline and D28 end of feeding period, each dog served as its own control, which controlled for inter-animal variability and allowed for the reporting of the food effect on a given metabolite.

Changes from the D0 baseline across foods for the global serum metabolome were evaluated with the Metaboanalyst platform v4.0 (78). Sparse partial least squares analysis (SPLS) distinguished among test food groups (number of components = 2, validation method = 5-fold cross-validation, number of predictors = 20). Random Forest detected metabolite predictors of food group identity (number of trees = 2,000, number of predictors = 20, Randomness = On).

Multivariate analysis of variance (MANOVA) was used to assess the degree to which a discrete class of metabolites was altered by food type. Initially, an interaction term (FO × MCT) was included in a two-way ANOVA model, but the results indicated that none of the FO × MCT interactions reached significance when corrected for false discovery rate (q > 0.1). Thereafter, the interaction term was omitted and a one-way ANOVA was used to determine a univariate group effect. Dependent sample (paired) *t*-tests on the D0 baseline vs. D28 end-of-feeding natural log-transformed values were used to determine whether the change from the D0 baseline for a particular group was different than zero for a given metabolite. Tukey's *post hoc* test determined which changes from the D0 baseline were different among the groups. All of these analyses were carried out in JMP (Version 14.2-15.0. SAS Institute Inc., Cary, NC, 1989–2019). Whether the change from the D0 baseline of a class of metabolites differed across the test food groups was determined by MANOVA using the identity function, which individually fits a model for each metabolite and subsequently tests the models together. Supplementary Table 4 shows MANOVA *p*-values for Wilks' lambda, Pillai's trace, Hotelling-Lawley, and Roy's Max Root. The metabolite class was considered impacted by food type only when *p*-values for all of these metrics were < 0.05. Two-way ANOVA in the Response Screening Platform with Cauchy robust fit in the JMP software package examined changes in individual metabolites within a class resulting from a dietary oil feeding study. In order to correct for multiple testing in the high-dimensional metabolomics data, ANOVA *p-*values were applied as an input, and q-values were generated for all metabolites (79) using the “qvalue” function in the R package qvalue v2.14.1 (80). Change in a metabolite was considered to be statistically significant when *p* ≤ 0.05 and q ≤ 0.1.

## 3. Results

### 3.1. Characteristics of dogs in the study

Sixty-four dogs (60 beagles and 4 mixed-breed) were randomized to one of four foods: CON, MCT, FO, or FO+MCT. The mean ± SE age was 5.9 ± 0.5 years, and the mean weight was 11.7 ± 0.4 kg; 41% were spayed females and 30% neutered males, while the rest were intact females (12%) and males (17%) (Supplementary Table 3). Following the study, all dogs were healthy and returned to the colony. There was no effect of food type on intake as quantified as kcal/kg BW^0.75^ and analyzed by a mixed model with an animal as a random factor for repeated intake measurements (*p* = 0.34).

### 3.2. Impact of dietary oils on clinical blood chemistry

Clinical blood chemistry values remained in normal ranges for healthy canines (Supplementary Table 4). FO consumption resulted in decreased total triglycerides from the D0 baseline (−8.31 mg/dL; *p* = 0.0453). FO+MCT also decreased triglycerides (−13.44 mg/dL; *p* < 0.0001), while MCT alone had no effect. Circulating cholesterol decreased from the D0 baseline in the FO group (−33.06 mg/dL; *p* = 0.0002) but increased in the MCT group (20.13 mg/dL; *p* = 0.0075). There was an overall decrease in cholesterol with FO+MCT feeding (−20.13 mg/dL; *p* = 0.0055).

### 3.3. Impact of dietary oils on the global serum metabolome

#### 3.3.1. Overview of metabolome

Metabolomic analysis performed on serum samples taken from dogs at the D0 baseline and week 4 identified 701 metabolites. Of these, one-way ANOVA showed that 354/701 metabolites (51%) were different across the four dietary groups (q-value FDR *p* ≤ 0.05, q ≤ 0.1). Individual dependent samples paired *t*-tests by food type to assess change relative to an individual's baseline indicated that 55/701 metabolites (8%) changed in the CON group from the D0 baseline values (31 increased, 24 decreased), 184/701 metabolites (26%) changed in the MCT group (59 increased, 125 decreased), 267/701 metabolites (38%) changed in the FO group (103 increased, 164 decreased), and 329/701 metabolites (47%) changed in the FO/MCT group (136 increased, 193 decreased) (Supplementary Table 4).

**Figure 1:**
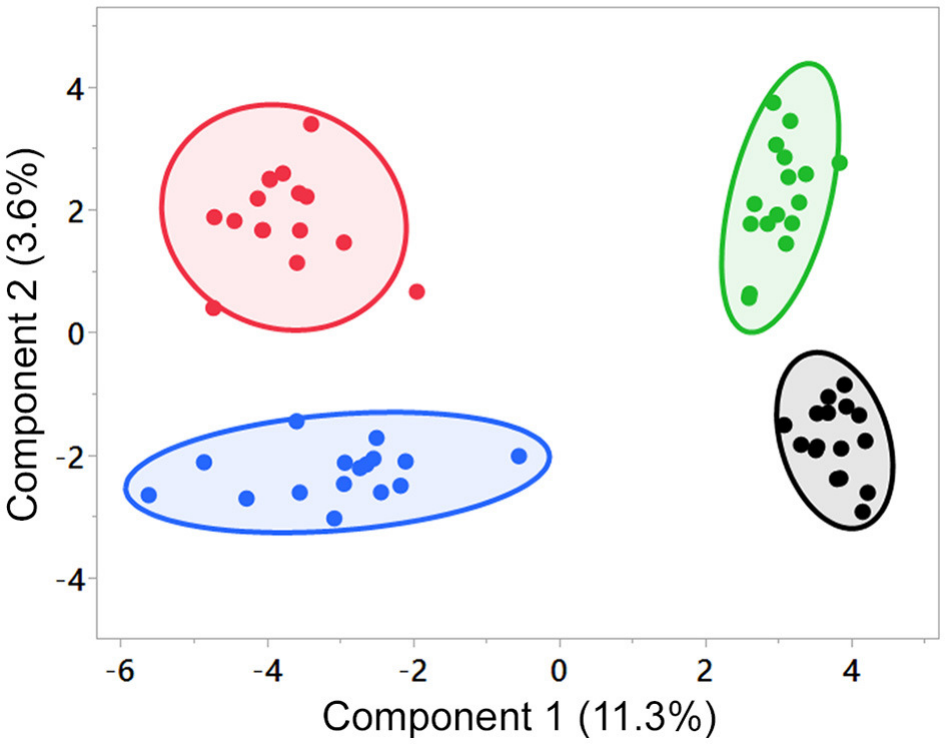
SPLS analysis of the test foods. Component 1 segregated groups by the presence of FO, and component 2 segregated groups by the presence of MCT in the food. Number of components = 2, validation method = 5-fold cross-validation, number of predictors = 20. Black, control group; green, MCT group; blue, FO group; red, FO+MCT group. Comp, component; FO, fish oil; MCTs, medium-chain fatty acid-containing triglycerides; SPLS, sparse partial least squares analysis.

SPLS indicated differences among the groups' changes from the D0 baseline (Figure 1), with no overlap of the 95% confidence regions among the foods; however, little of the variation was explained by the first two components (component 1, 11.3%; component 2, 3.6%). SPLS loadings for components 1 and 2 are in Supplementary Table 4. Random Forest analysis (Supplementary Figure 1) provided discrimination between the dietary groups with an overall out-of-bounds class error of 4.7%, where 16/16 dogs were correctly assigned to the control group (class error 0%), 14/16 dogs were correctly assigned to the MCT group (class error 12.5%), 15/16 dogs were correctly assigned to the FO group (class error 6.3%), and 16/16 dogs were correctly assigned to the FO+MCT group (class error 0%). As expected, the top 20 ranked Random Forest predictors were lipid species. Among both SPLS and Random Forest, the lipid species fed in the foods were predominant predictors of a food group membership. The first component of the SPLS was composed of FO-derived DHA- and EPA-containing complex lipids along with arachidonoyl-containing lipids and was indicative of dogs that had been fed FO (or FO+MCT). Accordingly, higher values on the second component indicated increased MCT-derived caproate (C6:0) and caprate (C10:0) and were indicative of MCT (or FO+MCT)-fed dogs.

Major lipid classes were chosen for further analysis: catabolic-type lipids [non-esterified fatty acids (NEFAs), mono- and diglycerides (MDAGs), acylcarnitines, alpha-oxidized fatty acids, omega-oxidized fatty acids (dioates)], signaling-type N-acyl amino acids/neurotransmitters (NAAN), structural-type complex lipids [glycerophosphatidylcholines (GPCs), glycerophosphatidylethanolamines (GPEs), glycerophosphatidylinositols (GPIs), and sphingolipids/ceramides (SPHING)], metabolites involved in central energy metabolism (amino acids, TCA cycle, methylation), and gut microbial postbiotics (indoles, phenols).

#### 3.3.2. Impact of MCT and FO on NEFAs

**Figure 2:**
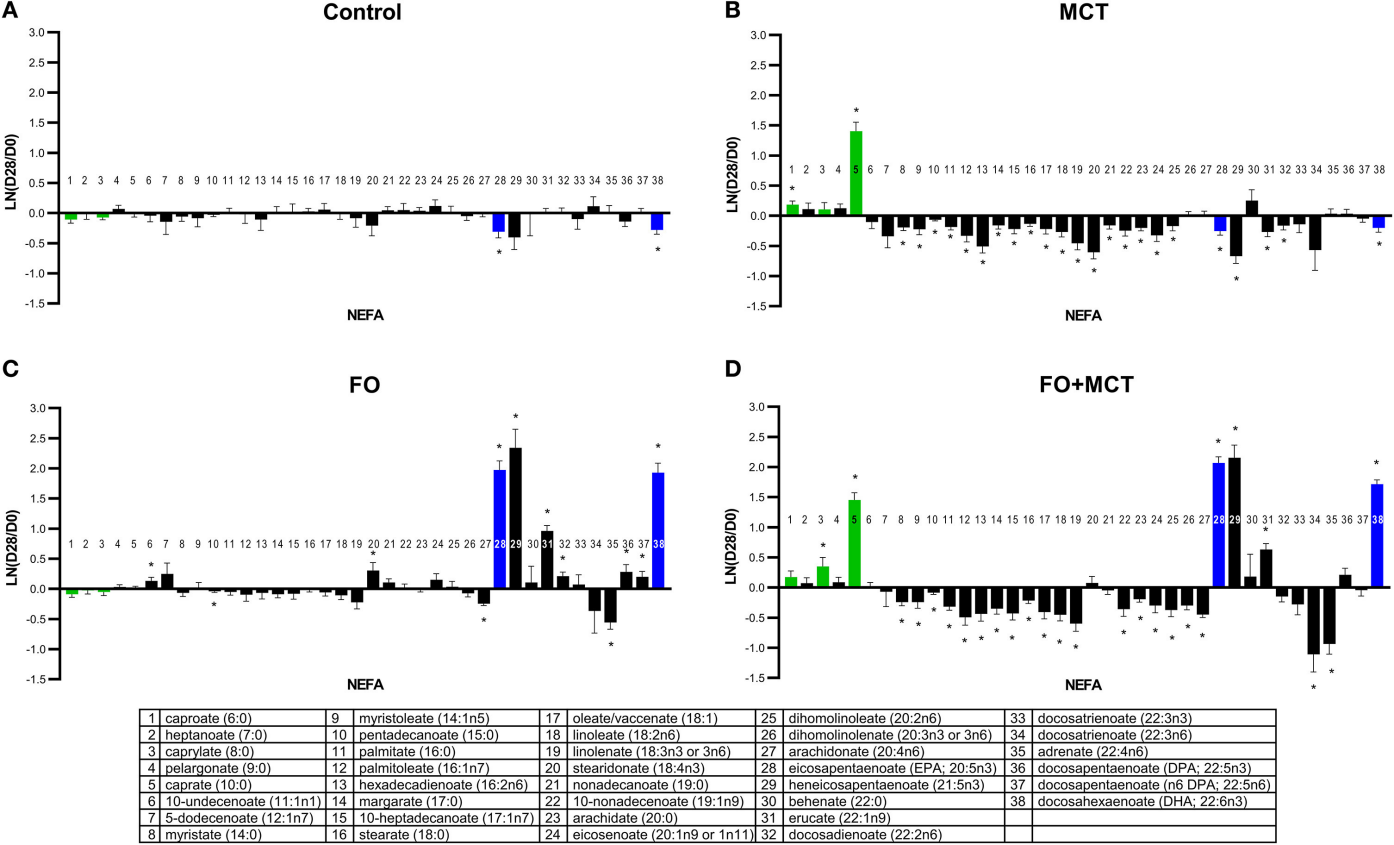
Change from the D0 baseline to D28 end of feeding period (LN fold change) in serum levels of non-esterified fatty acids. Dogs were fed **(A)** control food, **(B)** food with MCT, **(C)** food with FO, or **(D)** food with FO+MCT. Lipid metabolites are presented in order of increasing chain length and, within a chain length, by increasing unsaturation. Green bars indicate fatty acids found in the MCT ingredient; blue bars indicate fatty acids in the FO ingredient. FO, fish oil; MCTs, medium-chain fatty acid-containing triglycerides. *p < 0.05 compared with the D0 baseline.

The metabolite class of NEFAs was different across groups in a multivariate manner (MANOVA *p* < 0.001; Supplementary Table 4), and 30/38 (79%) of the observed NEFA changes from the D0 baseline were different across foods by univariate ANOVA (median *p* = 0.0035). The CON group remained largely unchanged from the D0 baseline while the MCT group exhibited decreases in several NEFAs, and the FO group showed increases in LCPUFA(n3) including DHA (22:6n3) (Figure 2). Feeding of FO+MCT gave largely the same as seen with MCT alone. Together, the MCT and FO+MCT groups showed reduced levels of most NEFA of carbon chain length of 14 through 20.

The medium-chain fatty acid (MCFA) caprate (C10:0) was different across groups by ANOVA (*p* ≤ 0.0001), and both the MCT and FO+MCT groups exhibited increased levels of caprate (C10:0) from the D0 baseline that were also different than changes seen in other groups. Another MCFA, caproate (C6:0), differed by food and increased from the D0 baseline in the MCT group, a change largely reproduced as a trend (*p* = 0.1002) in the combination of FO+MCT. The remaining MCFA, caprylate (C8:0), was different across the foods, driven largely by an increase solely in the FO+MCT group. The changes from the D0 baseline for the transition fat myristate (C14:0) were not different across the food groups. However, there was a decrease from the D0 baseline in this fatty acid in the MCT and FO+MCT groups.

Food type strongly altered the following LCPUFA(n3): stearidonate (18:4n3), EPA (20:5n3), heneicosapentaenoate (21:5n3), docosapentaenoate (DPA, 22:5n3), and DHA (22:6n3). These increased from the D0 baseline in the FO group; EPA (20:5n3), heneicosapentaenoate (21:5n3), and DHA (22:6n3) also increased in the FO+MCT group.

ARA (C20:4n6), a precursor to lipid signaling mediators including prostaglandins, thromboxanes, and leukotrienes, was decreased by the LCPUFA(n3)-containing foods (FO, FO+MCT). Adrenate (22:4n6), the elongation product of ARA (C20:4n6), was also decreased in the FO and FO+MCT groups. In contrast, both docosadienoate (22:2n6) and docosapentaenoate (n6 DPA; 22:5n6) were increased in the FO group but not in the FO+MCT group. The MCT group showed decreased levels of the n6 NEFA, including hexadecadienoate (16:2n6), linoleate (18:2n6), dihomolinoleate (20:2n6), and docosadienoate (22:2n6).

#### 3.3.3. Impact of MCT and FO on fatty acid glycerides, carnitines, endocannabinoid amides, and oxidation products

**Figure 3:**
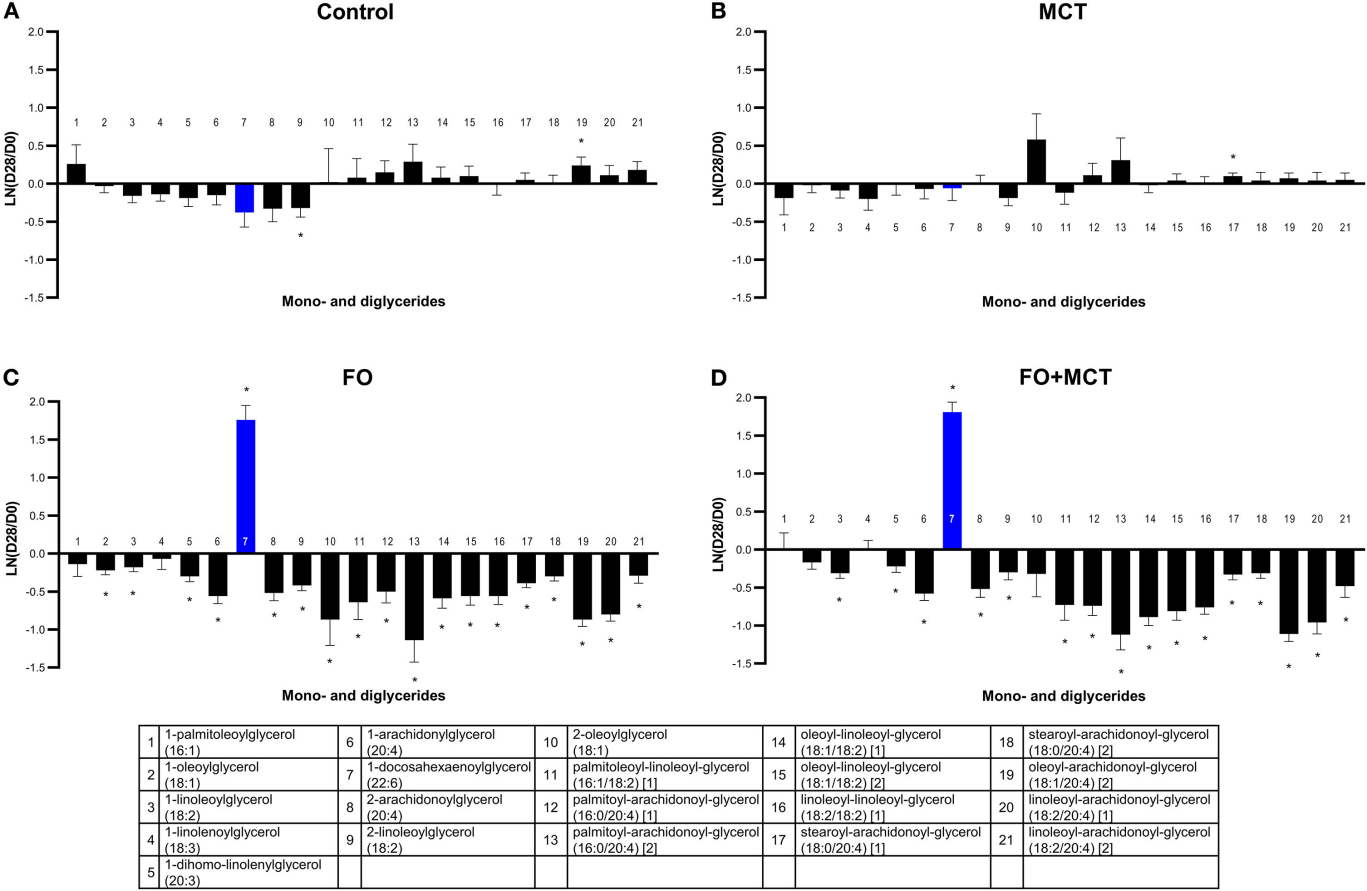
Change from the D0 baseline to D28 end of feeding period (LN fold change) in serum levels of mono- and diglycerides. Dogs were fed **(A)** control food, **(B)** food with MCT, **(C)** food with FO, or **(D)** food with FO+MCT. Blue bars indicate fatty acids in the FO ingredient. FO, fish oil; MCTs, medium-chain fatty acid-containing triglycerides. *p < 0.05 compared with the D0 baseline.

Multivariate analysis indicated that the MDAG class as a whole was changed by food type (MANOVA *p* < 0.0001; Supplementary Table 4). There were 21 metabolites detected in the data set, and of these, 16/21 (76%) were altered by food according to ANOVA (median *p* = 0.0002; Figure 3). The greatest effect was in the dogs consuming FO-containing foods; 19/21 (90%) of the MDAG were changed, and nearly all of these changes were decreases (1 up, 18 down). The FO+MCT group also had several changes in MDAG: 17/21 (81%) changed (1 up, 16 down). There was only 1/21 (5%) MDAG changed from the D0 baseline in the MCT group and 2/21 (10%) in the CON group.

**Figure 4:**
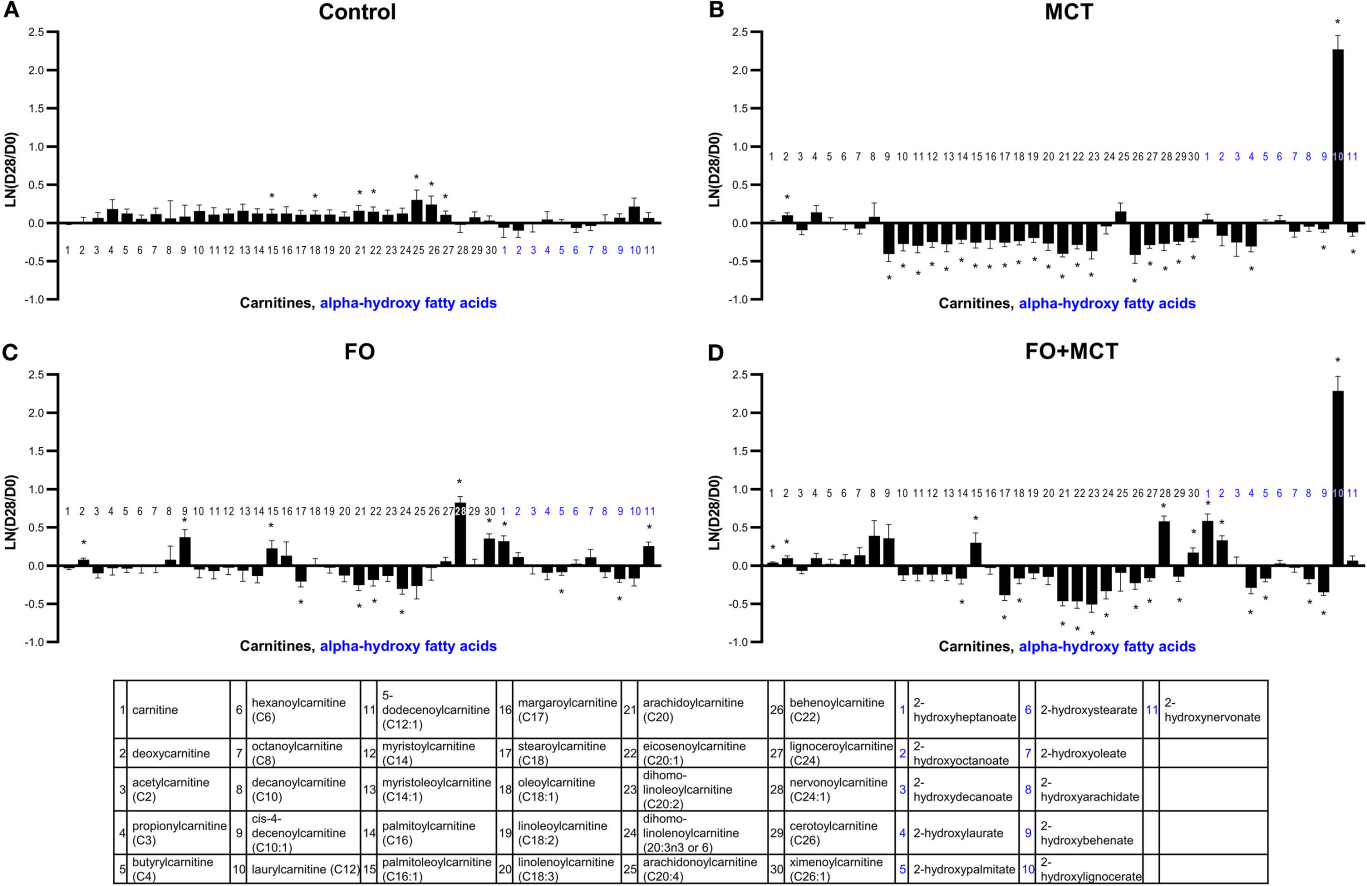
Change from the D0 baseline to D28 end of feeding period (LN fold change) in serum levels of carnitines and alpha-hydroxy fatty acids. Dogs were fed **(A)** control food, **(B)** food with MCT, **(C)** food with FO, or **(D)** food with FO+MCT. FO, fish oil; MCTs, medium-chain fatty acid-containing triglycerides. *p < 0.05 compared with the D0 baseline.

Acylcarnitines, including carnitine and deoxycarnitine as a multivariate class, were different by food group (MANOVA *p* < 0.0001; Supplementary Table 4). ANOVA detected 20/30 (67%) individual carnitines (median *p* = 0.0062; Figure 4 MCT alone decreased several acylcarnitines (Figure 4), with 21/30 (70%) changed (20 acylcarnitines decreased and deoxycarnitine increased). FO had a more moderate effect on acylcarnitines, with a similar mixture of up (5) and down (4) shifts from the D0 baseline (9/30 changed; 30%). The FO+MCT group manifested 5 increased and 10 decreased (15/30 changed; 50%). Arachidonoylcarnitine was not affected by food, and neither FO nor FO+MCT showed changes from the D0 baseline in this ARA (C20:4n6) metabolite. Deoxycarnitine, precursor to carnitine and acylcarnitines, was increased in all three experimental groups: MCT, FO, and FO+MCT. Furthermore, carnitine itself was increased from the D0 baseline in only the FO+MCT group, while it was unchanged in the MCT and FO groups.

**Figure 5:**
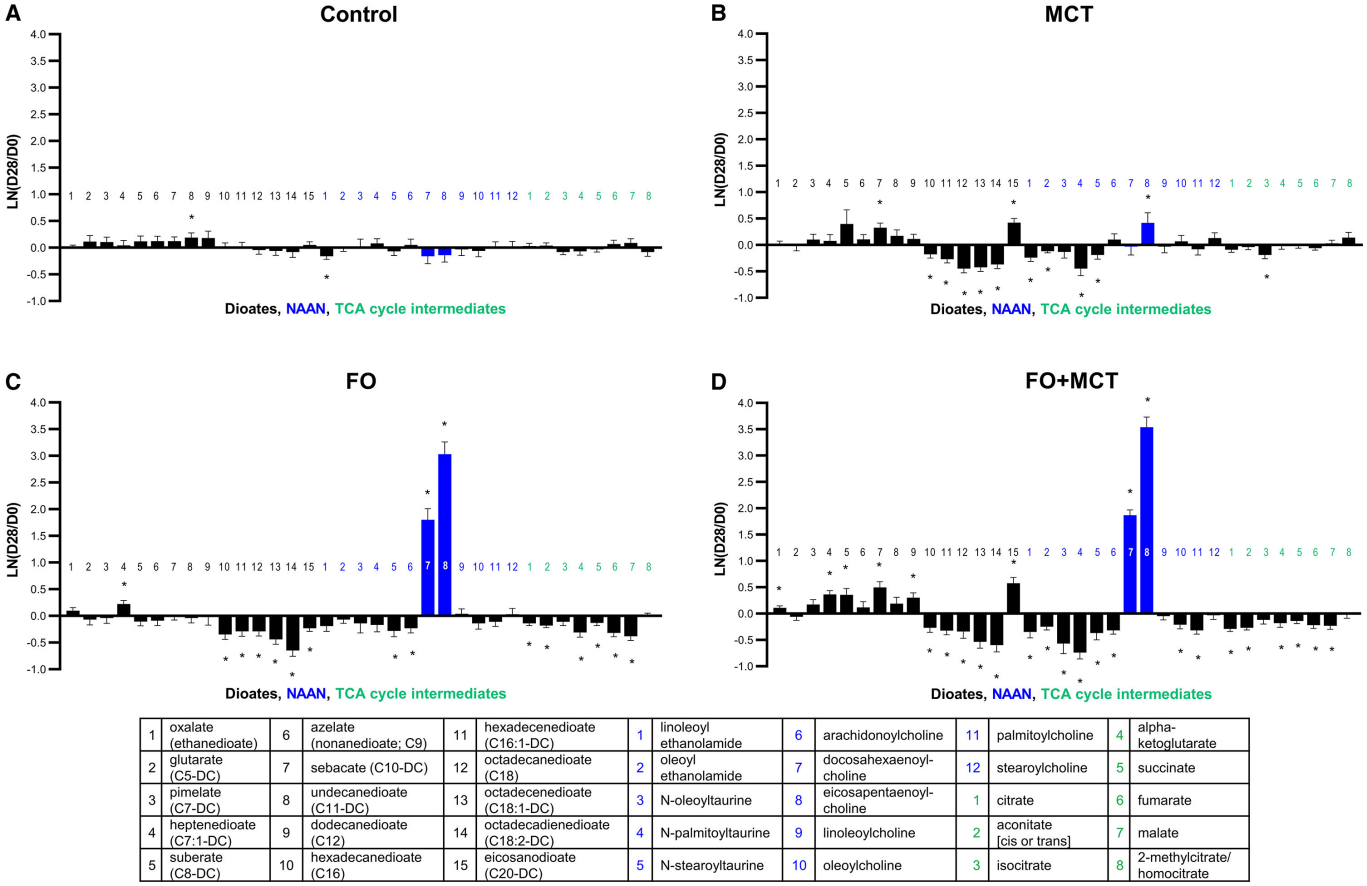
Change from the D0 baseline to D28 end of feeding period (LN fold change) in serum levels of dioates, NAAN, and TCA cycle intermediates. Dogs were fed **(A)** control food, **(B)** food with MCT, **(C)** food with FO, or **(D)** food with FO+MCT. Blue bars indicate fatty acids derived from the FO ingredient. FO, fish oil; MCTs, medium-chain fatty acid-containing triglycerides. NAAN, N-acyl amino acids/neurotransmitters; TCA, tricarboxylic acid. *p < 0.05 compared with the D0 baseline.

The serum metabolomics dataset yielded acylated amides from ethanolamide (*n* = 2), taurine (*n* = 3), and choline (*n* = 7), together the NAAN class. As a class, NAAN was different by MANOVA (*p* < 0.0001) and 7/12 (58%) individual NAAN were different by ANOVA (median *p* = 0.0129; Supplementary Table 4). The most changes to members of the NAAN class were observed in the FO+MCT group (10/12, 83%) compared with MCT (5/12, 42%) or FO (4/12, 33%) alone (Figure 5). All changes to NAAN not containing either DHA (C22:6n3) or EPA (C20:5n3) were decreases. Only NAAN containing either DHA (C22:6n3) or EPA (C20:5n3) were increased with FO or MCT.

Alpha-oxidized products of monocarboxylic fatty acids (AHFA) are considered to be generated in the endoplasmic reticulum and are precursors of sphingolipids/ceramides. The class of AHFA was different by food type (MANOVA *p* < 0.0004), and 8/11 (73%) of the individual AHFA were different by ANOVA (median *p* < 0.0001; Supplementary Table 4). Both MCT and FO+MCT feeding led to a multifold increase in 2-hydroxylignocerate (C24:0) and a concurrent decline in its unsaturation product 2-hydroxynervonate (C24:1) as well as its chain-shortened congener 2-hydroxybehenate (C22:0).

Dicarboxylate fatty acids (dioates) are produced by omega oxidation of the terminal carbon of monocarboxylic fatty acids in peroxisomes; this metabolite class was different by food type (MANOVA *p* < 0.0001) with 9/15 (60%) of the individual dioates driving this effect by ANOVA (median *p* = 0.0250; Figure 5). When FO and MCT were fed together, there were changes to 11/15 (73%) of dioates, with several changes to dioates of 12 or fewer carbons. Feeding with MCT and FO individually each produced changes in 7/15 (47%) of dioates, with the effect less evident in the dioates of 12 or fewer carbons.

Alternate forms of oxidized fatty acids were assessed as well. The products of fatty acid beta oxidation (3-hydroxy fatty acids) formed in mitochondria and the products of membrane oxidation (9- and 13-hydroxyoctadecadienoate [9-HODE and 13-HODE], 9,10-dihydroxy-12Z-octadecenoate [9,10-DiHOME], 12,13-dihydroxy-9Z-octadecenoate [12,13-DiHOME], 12-hydroxyeicosatetraenoate [12-HETE], 12-hydroxyheptadecatrienoate [12-HHTrE]) were not different as classes by food type, and there were no significant between-group differences (Supplementary Table 4).

#### 3.3.4. Impact of MCT and FO on phospholipids

**Figure 6:**
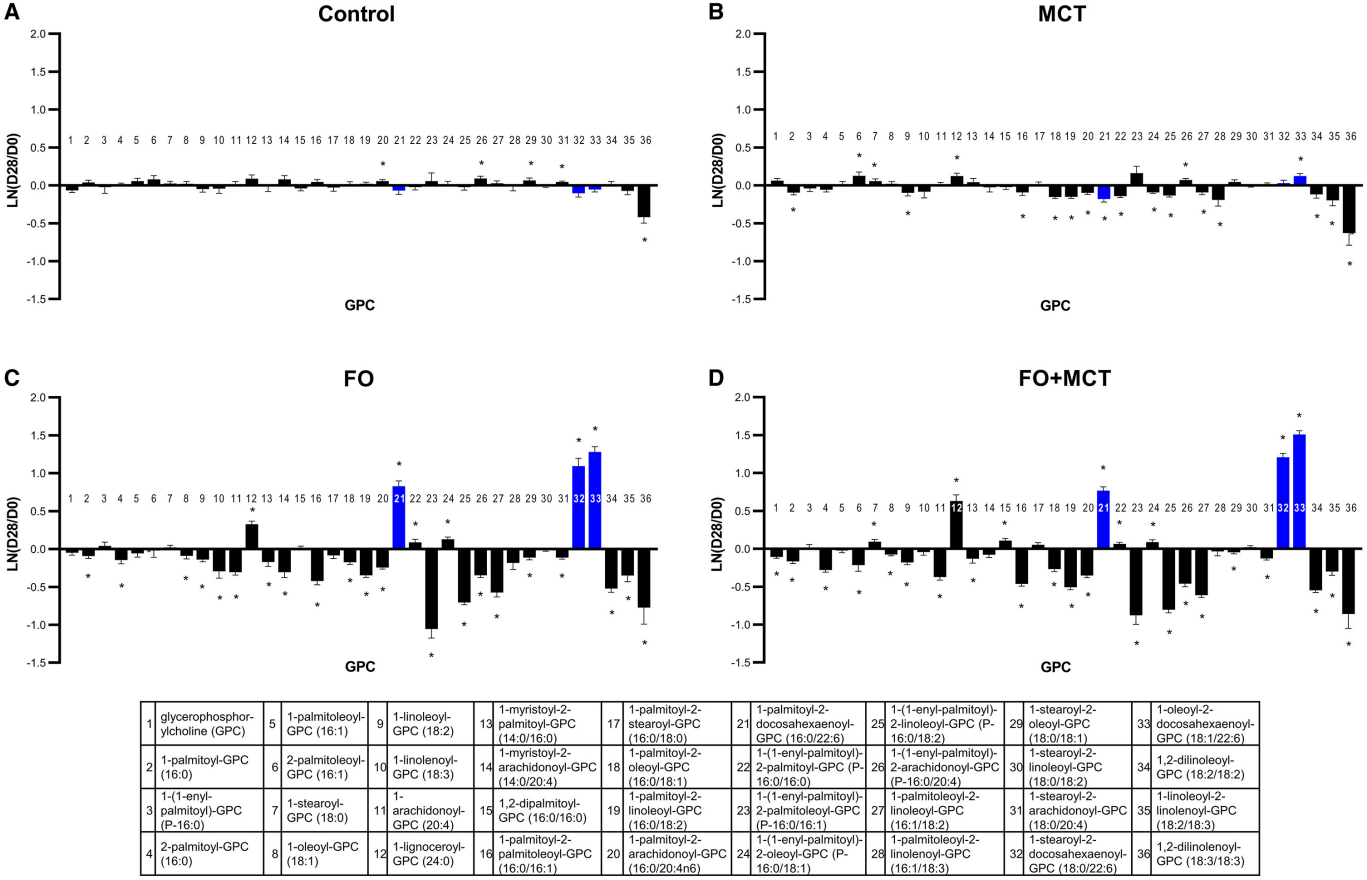
Change from the D0 baseline to D28 end of feeding period (LN fold change) in serum levels of GPCs. Dogs were fed **(A)** control food, **(B)** food with MCT, **(C)** food with FO, or **(D)** food with FO+MCT. Blue bars indicate fatty acids derived from the FO ingredient. FO, fish oil; GPC, glycerophosphatidylcholine; MCTs, medium-chain fatty acid-containing triglycerides. *p < 0.05 compared with the D0 baseline.

The metabolite class of GPCs was different across food groups in a multivariate manner (MANOVA *p* < 0.0001; Supplementary Table 4). Generally, GPCs were broadly affected by FO and/or MCT in that 29/36 (81%) of observed GPC changes from the D0 baseline were different across the groups (ANOVA median *p* < 0.0001; Figure 6). Choline differed by food group, and the MCT and FO+MCT groups had increased choline levels relative to the D0 baseline. Trimethylglycine was increased from the D0 baseline in the MCT group and decreased in the FO group (Supplementary Table 4). There was a decrease from the D0 baseline of glycerophosphorylcholine in only the FO+MCT group. FO alone, and to a lesser extent MCT, decreased GPCs; with FO feeding, 27/36 (75%) GPC changed (21/36 [58%] decreased) while with MCT feeding, 20/36 (56%) GPC changed (15/36 [42%] decreased). With the FO+MCT group, 29/36 (81%) GPC changed, with 21/36 (58%) decreased in the dogs receiving the combined oils. In all instances, GPCs with ARA (C20:4n6) inclusion at the sn-2 position were decreased by FO feeding. Furthermore, all except 1-myristoyl-2-arachidonoyl-GPC (14:0/20:4) were decreased in the FO+MCT group as well, indicating that the effect of FO to decrease ARA-GPC was preserved in the presence of MCT. FO feeding also led to increased DHA (C22:6n3) at the sn-2 position.

**Figure 7:**
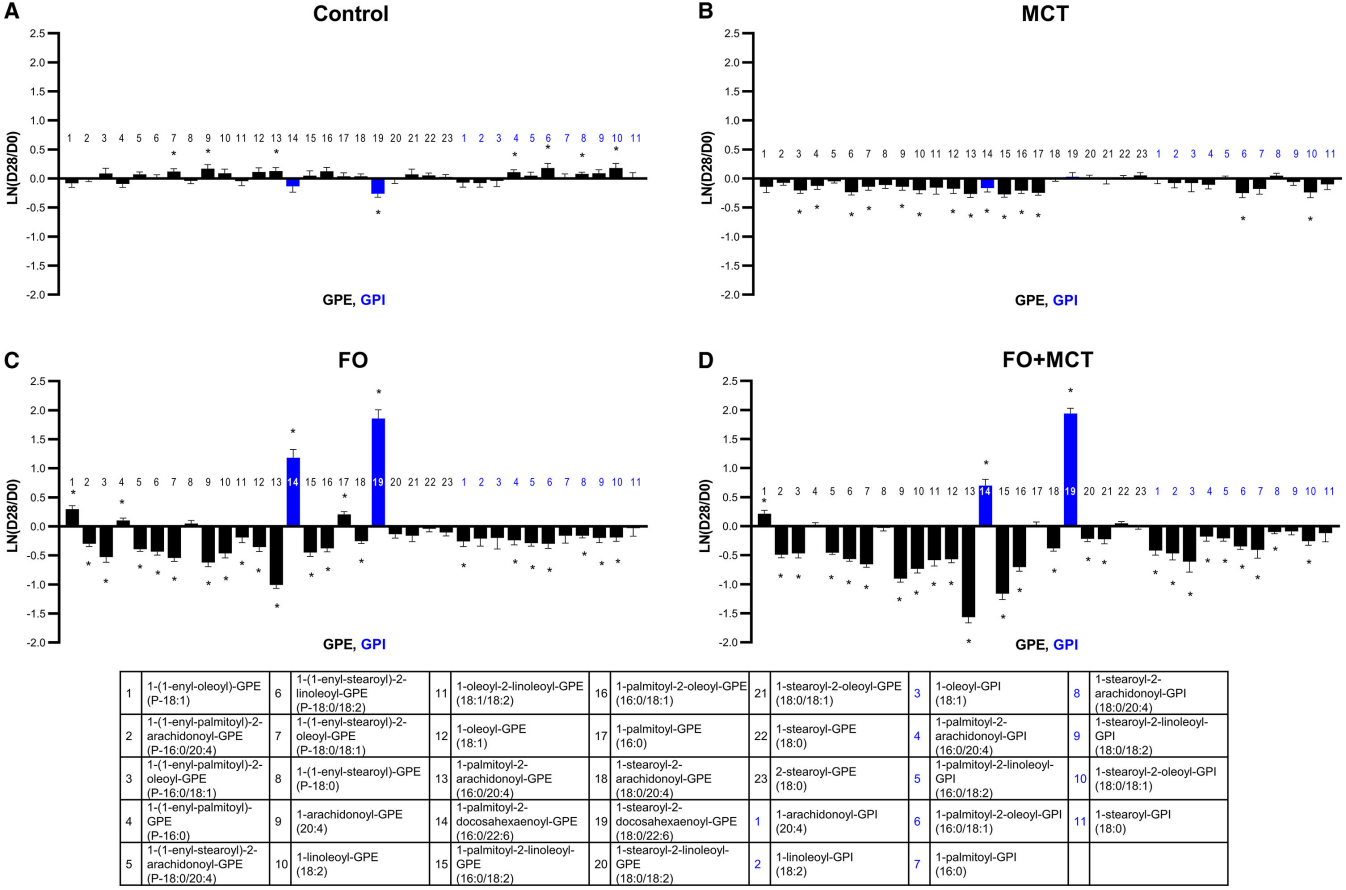
Change from the D0 baseline to D28 end of feeding period (LN fold change) in serum levels of GPEs and GPIs. Dogs were fed **(A)** control food, **(B)** food with MCT, **(C)** food with FO, or **(D)** food with FO+MCT. Blue bars indicate fatty acids derived from the FO ingredient. FO, fish oil; GPE, glycerophosphatidylethanolamine; GPI, glycerophosphatidylinositols; MCTs, medium-chain fatty acid-containing triglycerides. *p < 0.05 compared with the D0 baseline.

The GPE phospholipids as a class were also impacted by food type (MANOVA *p* < 0.0001), and 18/23 univariate changes among these GPE appeared to be the drivers (ANOVA median *p* < 0.0001; Figure 7; Supplementary Table 4). As with GPC, the predominant effect was for both FO and MCT individually to decrease GPE with the effect of FO being greater (18/23 [78%] changed with FO; 12/23 [52%] changed with MCT). Regarding ARA (C20:4n6)-containing GPE, both FO and FO+MCT decreased all five of these species observed in the dataset. As well, FO and FO+MCT increased both observed DHA (C22:6n3)-containing GPE lipids.

The GPI class of phospholipids was also different according to food group (MANOVA *p* < 0.0001), with 9/11 (82%) of these changed according to individual ANOVA (median *p* < 0.0001; Figure 7; Supplementary Table 4). The results observed for GPI were largely similar to those observed for GPC and GPE. FO had a greater effect to decrease a number of GPI than did MCT (FO changed 7/11 [64%] while MCT changed 2/11 [18%]), while FO+MCT presented the same pattern of changes as FO (9/11 changed; 82%). The phospholipid 1-palmitoyl-GPI (16:0) was not changed from the D0 baseline in either the FO or MCT groups (MCT, *p* = 0.0587; FO, *p* = 0.260) but was decreased in the FO+MCT group (*p* = 0.0096). GPI substituted at the sn-2 position with ARA (C20:4n6) were decreased in the FO and FO+MCT foods. No DHA (C22:6n3)- or EPA (C20:5n3)-containing GPI were detected.

**Figure 8:**
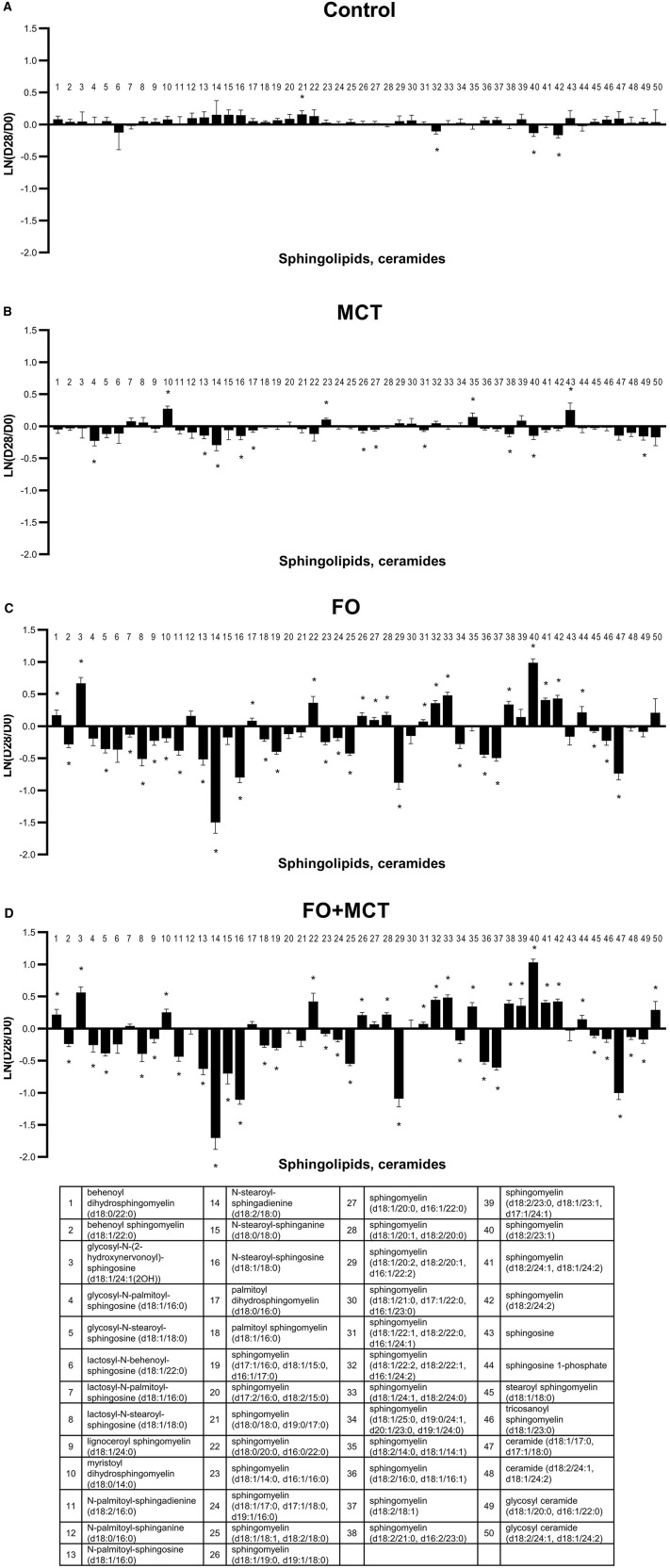
Change from the D0 baseline to D28 end of feeding period (LN fold change) in serum levels of sphingolipids and ceramides. Dogs were fed **(A)** control food, **(B)** food with MCT, **(C)** food with FO, or **(D)** food with FO+MCT. FO, fish oil; MCTs, medium-chain fatty acid-containing triglycerides. *p < 0.05 compared with the D0 baseline.

As a class, SPHING was different by group (MANOVA *p* = 0.0001); 39/50 (78%) SPHING were individually different by ANOVA across the food types (median *p* < 0.0001). While FO produced changes in 37/50 (74%) SPHING, MCT only generated changes from the D0 baseline for 15/50 (30%) and FO+MCT feeding resulted in 41/50 (82%) changes (Figure 8; Supplementary Table 4). For the N-palmitoyl and N-stearoyl series, an increasing degree of unsaturation of the SPHING moiety (sphinganine → sphingosine → sphingadienine) resulted in more strongly reduced levels with FO and FO+MCT treatments. Thus, unsaturated N-palmitoyl and N-stearoyl sphingosine and sphingadienine were decreased by FO consumption, while saturated sphinganine was not. Sphingosine-1-phosphate was increased by FO and FO+MCT foods but not by MCT treatment.

#### 3.3.5. Impact of MCT and FO on products of central metabolism

TCA cycle intermediates were found to be different as a class of metabolites by group (MANOVA *p* = 0.0003), with 4/8 (50%) of the TCA metabolites individually different by food type (ANOVA median *p* = 0.0533; Figure 5). While there was only one change in a TCA metabolite in the MCT group (decreased isocitrate), 6/8 (75%) TCA metabolites were changed in both the FO and FO+MCT group; all were decreased.

**Figure 9:**
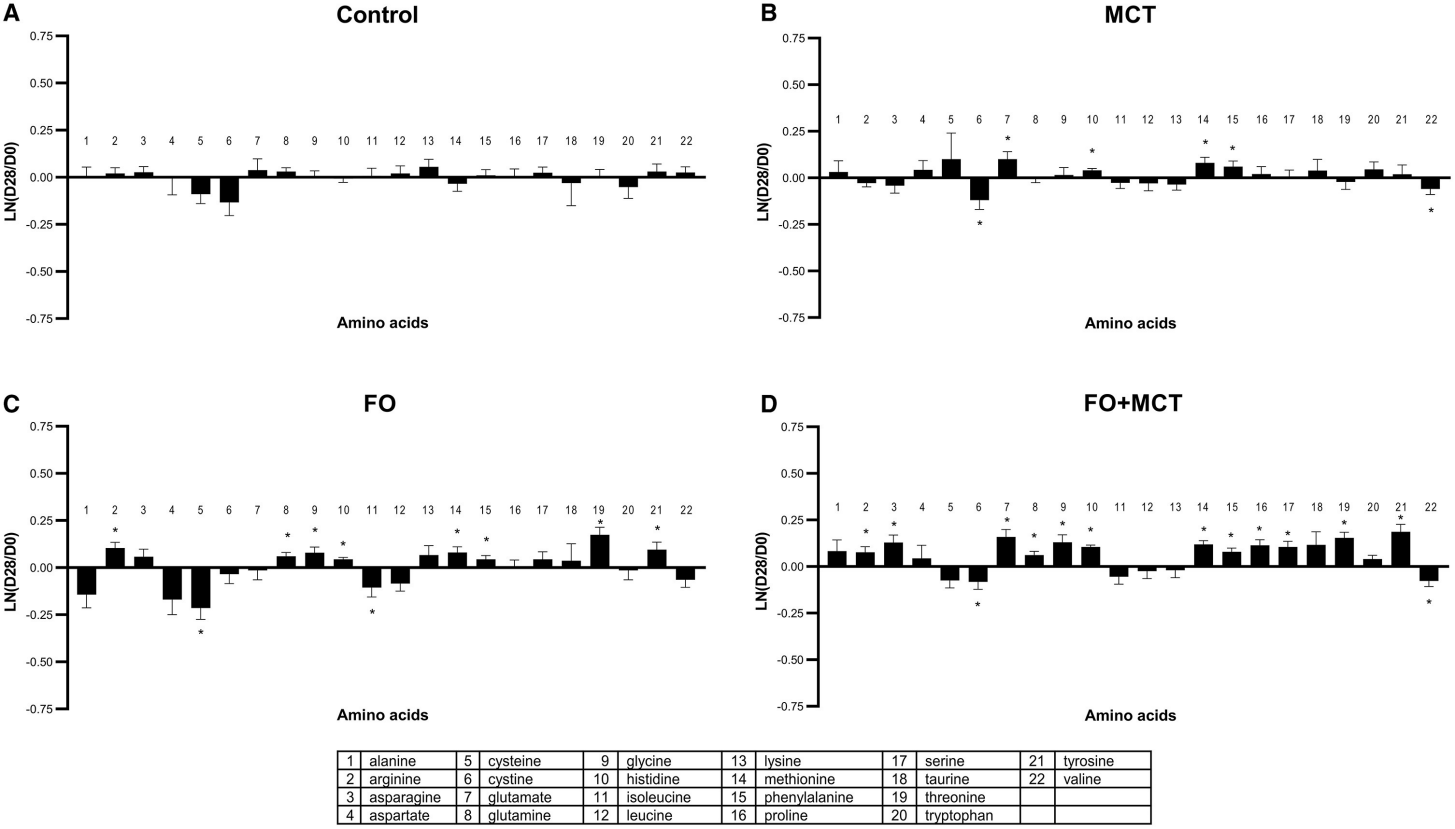
Change from the D0 baseline to D28 end of feeding period (LN fold change) in serum levels of amino acids. Dogs were fed **(A)** control food, **(B)** food with MCT, **(C)** food with FO, or **(D)** food with FO+MCT. FO, fish oil; MCTs, medium-chain fatty acid-containing triglycerides. *p < 0.05 compared with the D0 baseline.

Given that amino acids traffic nitrogen as well as carbon for energy, the levels of amino acids in response to feeding experimental foods were assessed. Proteogenic amino acids (plus taurine) as a class were different by group (MANOVA *p* < 0.0001), with 9/22 (41%) amino acids changed by food type according to ANOVA (median *p* = 0.0890; Figure 9; Supplementary Table 4). Most of the changes were present in the FO group (10/22; 45%), with fewer in the MCT group (6/22; 27%); these individual effects were compounded such that 14/22 (64%) amino acids were changed in the combination food FO+MCT. Most of these changes were increases, with only two amino acids decreased in each of the three experimental groups and the rest being increased circulating levels of amino acids.

#### 3.3.6. Impact of MCT and FO on microbial postbiotics

The metabolite class of indoles and indolic sulfates was different across groups in a multivariate manner with 16 total metabolites detected (MANOVA *p* < 0.0050; Supplementary Table 4), 5/16 (31%) of which were altered by food type (ANOVA). The MCT group exhibited changes in 2 indoles, with 3-indoxyl sulfate decreased but 7-hydroxyindole sulfate increased. Similarly, the FO group decreased 5-hydroxyindole sulfate but increased indolepropionate. The FO+MCT group showed changes in two indoles as well, both of which were decreased (5-, and 6-hydroxyindole sulfates).

The class of phenols and phenolic sulfates was different across groups by multivariate analysis (MANOVA *p* < 0.0005; Supplementary Table 4), with 25 total metabolites detected and 8/25 (32%) altered by food (ANOVA). Two additional phenol postbiotic metabolites showed food effect *p-*values ≤ 0.0800 and q-values ≤ 0.100. The control group presented with two phenols decreased from the D0 baseline: 3-acetylphenol sulfate and 4-vinylphenol sulfate. The MCT group exhibited only a single changed phenol, with 2-aminophenol sulfate increased. The FO group manifested six decreased phenols, including 4-allylphenol sulfate, 4-aminophenol sulfate, 4-ethylphenyl sulfate, 4-hydroxyphenylpyruvate, 4-vinylphenol sulfate, and phenylacetylalanine. The FO+MCT group showed the same changes as the FO alone group, with the exception that neither 4-hydroxyphenylpyruvate nor phenylacetylalanine changed in the FO+MCT group.

## 4. Discussion

This trial evaluated the effects of the addition of MCT and FO to food via global metabolomics to characterize the serum levels of several classes of energetic, structural, and signaling lipid metabolites. Changes in central metabolites, including those from the TCA cycle and amino acids, were analyzed to gain insight into the degree to which the dietary fats impact associated energetic pathways. Serum samples were drawn from 23-h fasted dogs. With regard to the metabolism of dietary fat, protein, and starch/sugar, available reports document that 23 h is sufficient time to become post-absorptive for dogs. The half-life of circulating triglycerides in dogs is 22 min, so dietary fat-derived fatty acids from a meal would no longer be circulating 23 h later but would instead be mobilized and trafficked from adipose stores or generated by hepatic *de novo* lipogenesis (81). In addition, blood urea nitrogen derived from postprandial amino acid catabolism returns to baseline between 16 and 24 h after a meal in dogs (82). For dietary carbohydrates, consumption of glucose leads to a return to baseline blood glucose levels approximately 90 min in dogs, while consumption of various starches leads to blood glucose returning to baseline by 3 h after a meal (83). Taken together, there is evidence that 23 h post-feeding can be considered a post-absorptive state in the dog.

In the current trial, dietary oil feeding in dogs led to reduced triglycerides. Consistent with trials in both dogs (66, 84) and humans (85), there was a decrease in circulating triglycerides with FO feeding. This decrease was also apparent in the FO+MCT group but not in the MCT group. The level of FO included was nearly 3% of the food dry matter in the FO and FO+MCT groups and replaced pork fat that was composed of ~35% saturated fat. Thus, the inclusion of FO led to an approximately 1% decrease in saturated fat in the FO and FO+MCT foods. Although decreasing dietary saturated fat is known to reduce circulating triglycerides in other species (86), it has been proposed that n3 fatty acid inclusion in the food can independently decrease triglycerides as well (86, 87). However, in contrast to the reports that indicate that FO can reduce triglycerides in dogs (66, 84), three FO feeding trials in dogs showed no effect of FO on triglycerides (36, 88, 89). The levels of total FO (or EPA + DHA) offered to dogs as well as the DHA-to-EPA ratio were lower in all of those trials than in the current trial. In the three trials that showed no effect of FO on triglycerides, one tested FO as a supplement in client-owned, mixed-breed dogs with an intake of 0.03–0.04 g FO/kg BW (88), while the other two studies used FO in food form at 0.11 g EPA+DHA/kg BW in Belgian Shepherd working dogs (89) and 0.06 g FO/kg BW in beagles (36). In the two trials that showed a reduction in triglycerides with FO, one tested FO as a supplement in miniature Schnauzers with primary hyperlipidemia (intake of 0.10 g EPA+DHA/kg BW, though it is unclear how this amount was present in one FO capsule) (84) and the other tested a food form of FO (0.10 g EPA+DHA/kg BW) in beagles (66). In the current study, the offering of FO to dogs was much higher (0.45 g FO/kg BW), and 0.19 g of EPA+DHA/kg BW was fed. As well, the ratio of DHA to EPA in the studies was 0.59 (88), 1.2 (89), 0.77 (84), 1.42 (66), 0.7 (36), and 7.3 in the currently reported trial. There is some evidence in humans that DHA more potently reduces triglycerides compared with EPA (90), although data on the effects of EPA vs. DHA on triglycerides in dogs are lacking. It may be that both the increased level of FO fatty acids and the increased ratio of DHA to EPA in the current report led to the observations that FO decreases triglycerides. The reason for the discordancy of results among previous reports, however, is not clear. It may be that breed or activity level was a determinant in those studies, although estimations of caloric intake and weight maintenance from these trials (where possible) indicate an activity factor of ~1.4–1.6 for all dogs. Another factor may be that when controlling for total fat level in the food (as was done in the current study), the inclusion of n3-rich FO will necessarily change fatty acid composition (68), a variable that cannot be deconvoluted in our study design. Thus, the ratio of FO-derived EPA and/or DHA to total fatty acids may be a determinant of the degree to which FO reduces triglycerides in dogs as well. In our previously published study in cats (38), the FO group also showed decreased triglyceride levels, although in that study the combination of FO+MCT did not decrease triglycerides, whereas it did in the present canine study. In the current study, consumption of MCT alone increased total cholesterol; in contrast, this lipid was decreased by FO feeding alone and FO+MCT. Indicative of the interaction of FO with MCT, the FO+MCT feeding was less potent in decreasing cholesterol than was FO alone, perhaps offset by the inclusion of MCT. Given that elevated triglycerides are associated with aging in dogs (49, 91), the current data add context to considerations that diets of older dogs are supplemented with sources of DHA and EPA such as FO.

There are few comparisons available for the canine circulating lipidome. Some publications examine non-serum matrices such as ocular fluids (60) or red blood cell membrane (65) and thus may not reflect circulating levels or include both structural and energetic lipids. Other publications have examined the impact of dietary oils on the circulating lipidome in dogs with inflammatory dermatological (64) or gastrointestinal (67) disease, and these disease states may perturb lipid metabolism relative to healthy canines of the sort enrolled in the current study. Some prior reports in this area of investigation have examined the changes in circulating lipid metabolites with LCPUFA and MCT feeding, but some of these studies did not include control foods (lacking both types of fats) or lacked a food that combined both fats (92–94). Previous publications have documented that feeding LCPUFA(n3) and MCFA-containing foods can increase the fatty acid, carnitine, and phospholipid fractions with these fats (17, 36, 38, 69). In the current trial, it was observed that consumption of LCPUFA(n3) and MCFA also enriched levels of these fats in the broader circulating lipidome. These phenomena included catabolic intermediates of MCT-derived fatty acids (e.g., the dioate sebacate [C10:0, MCT]) and signaling-type lipids derived from FO LCPUFA(n3) (e.g., docosahexenoylcholine [C22:6n3, FO]). This current report is novel in the factorial design of the oil feedings (alone or in combination) and the extensive reporting of the canine lipidome including structural, energetic, and signaling lipids.

When dietary levels of LCPUFA(n3) are increased, these fats increase at the sn-2 position of structural-type lipid classes including phospholipids, while LCPUFA(n6) fatty acids decrease at this position (24, 95). That phenomenon was particularly evident here with FO, which decreased phospholipids with an ARA (C20:4n6) at the sn-2 position. FO also decreased ARA (C20:4n6) as a free fatty acid (NEFA), ARA-containing MDAG, and NAAN arachidonoylcholine. Concurrently, FO also increased levels of DHA in phospholipids at the sn-2 position. A recent publication documented the impact of FO on circulating lipids in the plasma and erythrocytes from active dogs and noted that provision of the LCPUFA(n3) increased the incorporation of these lipids into phospholipids from both matrices at the expense of LCPUFA(n6) incorporation (89). When the total fatty acid makeup of serum was examined in healthy dogs after consumption of diets high in C18:2n6 or C18:1n9, the fatty acid contingent of the circulating lipidome was enriched in these respective fats (96). In a separate study, dogs consuming sources of two types of n3 fatty acids, linoleate (C18:3n3) from flaxseed oil, and EPA + DHA (C20:5n3 + C22:6n3) from FO, had increased n3 fatty acid content in the phospholipid fraction of the circulating lipidome (66). In the current study, the acylcarnitine derivative of ARA was not changed by FO or FO+MCT, indicating that increased beta oxidation of ARA to energy did not lead to the observed decreases in ARA and ARA-containing lipids. The levels of dietary ARA were not meaningfully different between the FO and CON groups, so differences did not arise from ARA intake variations. It appears then that the current study is consistent with a model of competitive interaction between n3-type and n6-type LCPUFA for incorporation into complex lipids. The fate of the decreased ARA is still lacking clarity; although circulating complex structural-type lipids manifested a decrease in ARA content, the energetic-type catabolic intermediate ARA-carnitine was not changed in compensation. ARA may be sequestered into a lipid fraction that is not in equilibrium with circulating lipids.

Considering energetic-type lipids in the post-absorptive canine subjects at the time of collection, the NEFA observed here were likely derived from lipolysis in adipose stores rather than from remnant circulating dietary fat. Although the dogs were increasingly reliant on fat for metabolism as glycogen stores are reduced over time without food, it is clear that they were not yet in ketosis. Circulating NEFA can be fated for energetic catabolism (direct beta oxidation in peripheral tissues or ketogenesis in the liver) or anabolic esterification processes that lead to phospholipids and triglycerides. Increased levels of NEFA (97) or acylcarnitines (98) are indicative of reduced capacity for central lipid metabolism. In the current study, MCT feeding produced greater decreases than did FO on energetic-type fatty acid metabolites. MCT led to changes in more end-stage catabolic products (e.g., NEFA and acylcarnitines) while FO impacted upstream intermediates of triglyceride catabolism (MDAG). It is unlikely that carnitine availability limited acylcarnitine levels in the MCT-fed dogs as carnitine was not changed by this oil. Interestingly, beta-oxidized fatty acids were not different as a class and there were few differences by diet. It may be that there was a decrease in NEFA and acylcarnitines without a concurrent increase in beta oxidation or that there was increased flux through beta oxidation without changes in levels of the members of this metabolite class. For non-lipid energetic and central metabolites, FO (with or without added MCT) showed broad changes to members of the TCA cycle class. In contrast, both FO and MCT individually and in combination impacted circulating amino acids. Omega oxidation is mostly carried out in the endoplasmic reticulum and produces dicarboxylic acids (dioates). It can occur when fatty acid beta oxidation is unable to keep pace with fatty acid flux (99). In the current trial, both FO and MCT feeding decreased all omega-oxidized C16 and C18 dioates. The MCFA (C6:0, C8:0, C10:0) are metabolized by microsomal cytochrome P45 enzymes to medium-chain dioates of the same chain length (100) and are reported to increase with MCT feeding in a manner distinct from that observed with fasting or abnormal fatty acid oxidation (101). The current results show that with MCT feeding sebacate dioate (C10:0) increased while suberate (C8:0) did not. Alpha-oxidized fatty acids can arise from peroxisomal oxidation of lipids involved in sphingolipid synthesis. In the current study, MCT feeding led to large increases in a single alpha-hydroxy fatty acid (2-hydroxylignocerate), which is incorporated into sphingomyelin that accumulates in the liver (102). The impact of FO and MCT feeding appeared to be minimal on HETE, HODE, HOME, and HHTrE-type lipid oxidation products, indicating some specificity of the actions of FO and MCT to impact structural, energetic, and some classes of signaling lipids. Taken together, it would appear that combined FO+MCT oil feeding may benefit metabolic status as indicated by improved lipid levels of NEFA, acylcarnitines, MDAG, omega and alpha-oxidized fatty acids, and non-lipid energetic intermediates.

Postbiotics are metabolites generated by gut microbial catabolism of food that bypassed small intestinal absorption. These metabolites can be absorbed by the host and appear in circulation to impact host physiology (103). Microbial putrefaction of tyrosine or tryptophan and phenylalanine produces the postbiotic phenols and indoles, respectively (104), and these can have negative effects on host health, including on renal function and inflammation (105). In the current study, no endpoints were measured from feces, and thus, the origin of the molecules observed in the circulating metabolomics dataset that are canonically considered postbiotics cannot unambiguously be known. However, some of the putative postbiotic molecules have been observed to change in blood in previous studies and assessment of their response to FO and/or MCT in dogs helps to inform design and criteria for future studies. It was recently published that food can decrease levels of potentially detrimental postbiotics in dogs (106), and the current findings indicate that the spectrum of foods that might decrease these postbiotics is broader than previously understood. In the previously published feline study (38), microbial putrefaction products of aromatic amino acids were decreased with combined FO+MCT feeding, although this was not evident to the same degree in the current study with canine subjects. In the current study, the foods produced changes in 32% of observed microbiome putrefactive postbiotics of both the indole and phenol classes. This is a less pervasive effect for canines than that observed in the feeding of these oils to felines, where 73% of observed indoles and 83% of observed phenols were altered by food type (38). Furthermore, whereas the FO+MCT condition provided a starkly evident effect for the FO+MCT combination to decrease postbiotics in cats, in the current study with dogs no such effect was apparent.

In most instances, the qualitative effects of FO or MCT alone appeared to be additive such that lipids appearing upon consumption of one oil would still be present at similar levels when the oils were fed together. For example, the signaling-type lipid sphingosine-1-phosphate (107) was increased by the FO-containing foods with no impact by MCT. Similarly, MCT appeared to not impact the effect of FO on TCA metabolites as both FO and FO+MCT groups responded similarly. This additive nature of the effects of FO and MCT was evident for some phospholipids. The lysophospholipid 1-(1-enyl-palmitoyl)-GPE (P-16:0) was decreased by MCT but increased by FO with the overall effect in the FO+MCT group being no change. The same was true for 1-palmitoyl-GPE (16:0); MCT decreased while FO increased levels and FO+MCT showed no change. In some cases, the combination of FO+MCT feeding produced more marked changes for energetic and signaling lipids than when either oil was fed alone, including alpha- and omega-oxidized fatty acids (AHFA and dioates) and the endocannabinoid NAAN class. On balance, though, there was little statistical interaction apparent for lipid metabolites.

We previously published the results of a similar feeding trial in cats noting the effects of FO and/or MCT on the same classes of lipids (38). This provides an opportunity to assess species differences in canine and feline responses to the same dietary levels of the same bioactive fats. As examples of species differences in responses, in dogs MCT had a broad effect to decrease several NEFA even when FO was fed at the same time (FO+MCT). In cats, however, while MCT feeding also broadly decreased NEFA, the combination of FO+MCT increased NEFA (38). Another dissimilarity in response was that in dogs MCT decreased acylcarnitines regardless of the presence of FO, while in cats this lipid class was largely not impacted by MCT feeding and was increased by FO+MCT (38). There was also some concordance in response of some lipid types in cats and dogs. Both the cat and dog studies showed that FO+MCT led to decreases in N-acyl taurines, the accumulation of which (along with acylcarnitines) appears to lead to beta-cell dysfunction and type 2 diabetes (108). There was extensive agreement in cat and dog responses for phospholipids (GPC, GPE) and sphingolipids; in both species, the patterns observed for these lipid classes with FO+MCT were remarkably similar to the pattern with FO alone. Also seemingly concordant between the cat and dog findings was the decrease in several members of the NAAN class of endocannabinoid signaling lipids with combination FO+MCT oil feeding that was less responsive to either individual oil alone.

A strength of this study was the longitudinal design, which allowed a comparison of the changes induced by oil feeding rather than only providing a cross-sectional post-feeding assessment. Furthermore, the 2 × 2 study design allowed for the assessment of the interaction between the feeding of MCT and FO. A limitation of this design, however, was that the study was not performed in a crossover or Latin square design due to the constraints perceived around the long-term carryover effects of oil feeding. Thus, as each dog only consumed one of the oil formulations throughout the study, a comparison of different oil formulations within each subject was not possible. A further limitation of this study was that feces were not collected, and thus, analysis of the source of putative circulating postbiotics is not possible; the current data are thus only minimally useful in drawing firm conclusions on the source of putative postbiotics and their response to the dietary oil feeding.

In summary, feeding dogs MCT, FO, or FO+MCT demonstrated responsiveness of several simple and complex lipid classes and characterized the patterns of metabolites that drove these class-wise changes. The current study provides valuable insights into canine physiology in response to feeding dietary oils that can be employed therapeutically (FO for mobility and MCT for seizures). In this trial, dogs responded to FO consumption with a reduction in triglycerides. Consumption of MCT largely led to changes in lipids associated with energy metabolism, while FO consumption produced changes dominated by structural-type lipids. Both of these observations are consistent with the known biology of these lipids, where MCT are employed for metabolic disease and FO can be employed to alter membrane fluidity and triglycerides. These data confirm previous reports that consumption of LCPUFA(n3) decreases the incorporation of LCPUFA(n6) into circulating phospholipid fractions. Taken together, it can be concluded that lipidomic signatures relevant to the clinical efficacy of FO or MCT are maintained when these oils are fed in combination, and this report provides insights into which classes of lipids are most responsive to either dietary oil.

## Data availability statement

The original contributions presented in the study are included in the article/Supplementary material, and further inquiries can be directed to the corresponding author.

## Ethics statement

The animal studies were approved by Institutional Animal Care and Use Committee, Hill's Pet Nutrition, Topeka, KS, USA. The studies were conducted in accordance with the local legislation and institutional requirements. Written informed consent was not obtained from the owners for the participation of their animals in this study because animals were owned by Hill's Pet Nutrition.

## Author contributions

MJ and DJ designed the project and methodology, acquired the funding and resources, curated the data, and performed the formal analysis. MJ wrote the first draft of the manuscript. MJ and DJ reviewed and edited the manuscript. All authors have read and agreed to the published version of the manuscript.

## References

[1] Wernimont SM Radosevich J Jackson MI Ephraim E Badri DV MacLeay JM. The effects of nutrition on the gastrointestinal microbiome of cats and dogs: impact on health and disease. Front Microbiol. (2020) 11:1266. 10.3389/fmicb.2020.01266 32670224 PMC7329990

[2] De Angelis M Ferrocino I Calabrese FM De Filippis F Cavallo N Siragusa S. Diet influences the functions of the human intestinal microbiome. Sci Rep. (2020) 10:4247. 10.1038/s41598-020-61192-y 32144387 PMC7060259

[3] Singh RK Chang HW Yan D Lee KM Ucmak D Wong K. Influence of diet on the gut microbiome and implications for human health. J Transl Med. (2017) 15:73. 10.1186/s12967-017-1175-y 28388917 PMC5385025

[4] Wang Z Koonen D Hofker M Fu J. Gut microbiome and lipid metabolism: from associations to mechanisms. Curr Opin Lipidol. (2016) 27:216–24. 10.1097/MOL.0000000000000308 27054442

[5] Zhou S Wang Y Jacoby JJ Jiang Y Zhang Y Yu LL. Effects of medium- and long-chain triacylglycerols on lipid metabolism and gut microbiota composition in C57BL/6J mice. J Agric Food Chem. (2017) 65:6599–607. 10.1021/acs.jafc.7b01803 28704610

[6] Zentek J Ferrara F Pieper R Tedin L Meyer W Vahjen W. Effects of dietary combinations of organic acids and medium chain fatty acids on the gastrointestinal microbial ecology and bacterial metabolites in the digestive tract of weaning piglets. J Anim Sci. (2013) 91:3200–10. 10.2527/jas.2012-5673 23798515

[7] Kono H Fujii H Asakawa M Yamamoto M Matsuda M Maki A. Protective effects of medium-chain triglycerides on the liver and gut in rats administered endotoxin. Ann Surg. (2003) 237:246–55. 10.1097/01.SLA.0000048450.44868.B1 12560783 PMC1522134

[8] Kono H Fujii H Ishii K Hosomura N Ogiku M. Dietary medium-chain triglycerides prevent chemically induced experimental colitis in rats. Transl Res. (2010) 155:131–41. 10.1016/j.trsl.2009.08.011 20171598

[9] Hanczakowska E Szewczyk A Okoń K. Effects of dietary caprylic and capric acids on piglet performance and mucosal epithelium structure of the ileum. J Anim Feed Sci. (2011) 20:556–65. 10.22358/jafs/66213/2011

[10] Lee SI Kang KS. Function of capric acid in cyclophosphamide-induced intestinal inflammation, oxidative stress, and barrier function in pigs. Sci Rep. (2017) 7:16530. 10.1038/s41598-017-16561-5 29184078 PMC5705592

[11] Ahlstrøm Ø Krogdahl A Vhile SG Skrede A. Fatty acid composition in commercial dog foods. J Nutr. (2004) 134:2145s−7s. 10.1093/jn/134.8.2145S 15284422

[12] Bauer JE. The essential nature of dietary omega-3 fatty acids in dogs. J Am Vet Med Assoc. (2016) 249:1267–72. 10.2460/javma.249.11.1267 27875089

[13] Cui C Li Y Gao H Zhang H Han J Zhang D. Modulation of the gut microbiota by the mixture of fish oil and krill oil in high-fat diet-induced obesity mice. PLoS ONE. (2017) 12:e0186216. 10.1371/journal.pone.0186216 29016689 PMC5633193

[14] Han J Cui C Lib Y Gaoc H Zhanga H Zhanga C. Dietary supplement with a mixture of fish oil and krill oil has sex-dependent effects on obese mice gut microbiota. J Funct Foods. (2018) 51:47–54. 10.1016/j.jff.2018.07.052

[15] Zhang D Han J Li Y Yuan B Zhou J Cheong L. Tuna oil alleviates D-galactose induced aging in mice accompanied by modulating gut microbiota and brain protein expression. J Agric Food Chem. (2018) 66:5510–20. 10.1021/acs.jafc.8b00446 29656644

[16] Zicker SC Jewell DE Yamka RM Milgram NW. Evaluation of cognitive learning, memory, psychomotor, immunologic, and retinal functions in healthy puppies fed foods fortified with docosahexaenoic acid-rich fish oil from 8 to 52 weeks of age. J Am Vet Med Assoc. (2012) 241:583–94. 10.2460/javma.241.5.583 22916855

[17] Hall JA Brockman JA Jewell DE. Dietary fish oil alters the lysophospholipid metabolomic profile and decreases urinary 11-dehydro thromboxane B(2) concentration in healthy beagles. Vet Immunol Immunopathol. (2011) 144:355–65. 10.1016/j.vetimm.2011.08.007 21925741

[18] Fritsch D Allen TA Dodd CE Jewell DE Sixby KA Leventhal PS. Dose-titration effects of fish oil in osteoarthritic dogs. J Vet Intern Med. (2010) 24:1020–6. 10.1111/j.1939-1676.2010.0572.x 20707845

[19] Roush JK Cross AR Renberg WC Dodd CE Sixby KA Fritsch DA. Evaluation of the effects of dietary supplementation with fish oil omega-3 fatty acids on weight bearing in dogs with osteoarthritis. J Am Vet Med Assoc. (2010) 236:67–73. 10.2460/javma.236.1.67 20043801

[20] Roush JK Dodd CE Fritsch DA Allen TA Jewell DE Schoenherr WD. Multicenter veterinary practice assessment of the effects of omega-3 fatty acids on osteoarthritis in dogs. J Am Vet Med Assoc. (2010) 236:59–66. 10.2460/javma.236.1.59 20043800

[21] Fritsch DA Allen TA Dodd CE Jewell DE Sixby KA Leventhal PS. A multicenter study of the effect of dietary supplementation with fish oil omega-3 fatty acids on carprofen dosage in dogs with osteoarthritis. J Am Vet Med Assoc. (2010) 236:535–9. 10.2460/javma.236.5.535 20187817

[22] Li K Huang T Zheng J Wu K Li D. Effect of marine-derived n-3 polyunsaturated fatty acids on C-reactive protein, interleukin 6 and tumor necrosis factor alpha: a meta-analysis. PLoS ONE. (2014) 9:e88103. 10.1371/journal.pone.0088103 24505395 PMC3914936

[23] Costantini L Molinari R Farinon B Merendino N. Impact of omega-3 fatty acids on the gut microbiota. Int J Mol Sci. (2017) 18:2645. 10.3390/ijms18122645 29215589 PMC5751248

[24] Jewell DE Jackson MI. Dietary betaine and fatty acids change circulating single-carbon metabolites and fatty acids in the dog. Animals (Basel). (2022) 12:768. 10.3390/ani12060768 35327165 PMC8944756

[25] Li Q Zhang Q Wang M Zhao S Xu G Li J. n-3 polyunsaturated fatty acids prevent disruption of epithelial barrier function induced by proinflammatory cytokines. Mol Immunol. (2008) 45:1356–65. 10.1016/j.molimm.2007.09.003 17936906

[26] Kaur N Chugh V Gupta AK. Essential fatty acids as functional components of foods- a review. J Food Sci Technol. (2014) 51:2289–303. 10.1007/s13197-012-0677-0 25328170 PMC4190204

[27] Popa I Solgadi A Pin D Watson AL Haftek M Portoukalian J. The linoleic acid content of the stratum corneum of ichthyotic golden retriever dogs is reduced as compared to healthy dogs and a significant part is oxidized in both free and esterified forms. Metabolites. (2021) 11:803. 10.3390/metabo11120803 34940561 PMC8704365

[28] Simopoulos AP. The importance of the ratio of omega-6/omega-3 essential fatty acids. Biomed Pharmacother. (2002) 56:365–79. 10.1016/S0753-3322(02)00253-6 12442909

[29] Abulizi N Quin C Brown K Chan YK Gill SK Gibson DL. Gut mucosal proteins and bacteriome are shaped by the saturation index of dietary lipids. Nutrients. (2019) 11:418. 10.3390/nu11020418 30781503 PMC6412740

[30] Berk BA Law TH Packer RMA Wessmann A Bathen-Nöthen A Jokinen TS. A multicenter randomized controlled trial of medium-chain triglyceride dietary supplementation on epilepsy in dogs. J Vet Intern Med. (2020) 34:1248–59. 10.1111/jvim.15756 32293065 PMC7255680

[31] Molina J Jean-Philippe C Conboy L Añor S de la Fuente C Wrzosek MA. Efficacy of medium chain triglyceride oil dietary supplementation in reducing seizure frequency in dogs with idiopathic epilepsy without cluster seizures: a non-blinded, prospective clinical trial. Vet Rec. (2020) 187:356. 10.1136/vr.105410 32532842 PMC7799411

[32] Berk BA Packer RMA Law TH Wessmann A Bathen-Nöthen A Jokinen TS. Medium-chain triglycerides dietary supplement improves cognitive abilities in canine epilepsy. Epilepsy Behav. (2021) 114:107608. 10.1016/j.yebeh.2020.107608 33268017

[33] Carlson SJ Nandivada P Chang MI Mitchell PD O'Loughlin A Cowan E. The addition of medium-chain triglycerides to a purified fish oil-based diet alters inflammatory profiles in mice. Metabolism. (2015) 64:274–82. 10.1016/j.metabol.2014.10.005 25458829 PMC4277814

[34] Baker MA Cho BS Anez-Bustillos L Dao DT Pan A O'Loughlin AA. Fish oil-based injectable lipid emulsions containing medium-chain triglycerides or added α-tocopherol offer anti-inflammatory benefits in a murine model of parenteral nutrition-induced liver injury. Am J Clin Nutr. (2019) 109:1038–50. 10.1093/ajcn/nqy370 30882140 PMC6462433

[35] Kondreddy VK Anikisetty M Naidu KA. Medium-chain triglycerides and monounsaturated fatty acids potentiate the beneficial effects of fish oil on selected cardiovascular risk factors in rats. J Nutr Biochem. (2016) 28:91–102. 10.1016/j.jnutbio.2015.10.005 26878786

[36] Hall JA Jewell DE. Feeding healthy beagles medium-chain triglycerides, fish oil, and carnitine offsets age-related changes in serum fatty acids and carnitine metabolites. PLoS ONE. (2012) 7:e49510. 10.1371/journal.pone.0049510 23145181 PMC3492282

[37] Li Q Heaney A Langenfeld-McCoy N Boler BV Laflamme DP. Dietary intervention reduces left atrial enlargement in dogs with early preclinical myxomatous mitral valve disease: a blinded randomized controlled study in 36 dogs. BMC Vet Res. (2019) 15:425. 10.1186/s12917-019-2169-1 31775756 PMC6882217

[38] Jackson MI Jewell DE. Docosahexaenoate-enriched fish oil and medium chain triglycerides shape the feline plasma lipidome and synergistically decrease circulating gut microbiome-derived putrefactive postbiotics. PLoS One. (2020) 15:e0229868. 10.1371/journal.pone.0229868 32163448 PMC7067441

[39] Hoffman JM Creevy KE Franks A O'Neill DG Promislow DEL. The companion dog as a model for human aging and mortality. Aging Cell. (2018) 17:e12737. 10.1111/acel.12737 29457329 PMC5946068

[40] Kleinert M Clemmensen C Hofmann SM Moore MC Renner S Woods SC. Animal models of obesity and diabetes mellitus. Nat Rev Endocrinol. (2018) 14:140–62. 10.1038/nrendo.2017.161 29348476

[41] Osto M Lutz TA. Translational value of animal models of obesity-Focus on dogs and cats. Eur J Pharmacol. (2015) 759:240–52. 10.1016/j.ejphar.2015.03.036 25814247

[42] Moore MC Menon R Coate KC Gannon M Smith MS Farmer B. Diet-induced impaired glucose tolerance and gestational diabetes in the dog. J Appl Physiol. (2011) 110:458–67. 10.1152/japplphysiol.00768.2010 21088210 PMC3043793

[43] Kwong LK Feingold KR Peric-Golia L Le T Karkas JD Alberts AW. Intestinal and hepatic cholesterogenesis in hypercholesterolemic dyslipidemia of experimental diabetes in dogs. Diabetes. (1991) 40:1630–9. 10.2337/diab.40.12.1630 1756903

[44] Pucheu-Haston CM Shuster D Olivry T Brianceau P Lockwood P McClanahan T. A canine model of cutaneous late-phase reactions: prednisolone inhibition of cellular and cytokine responses. Immunology. (2006) 117:177–87. 10.1111/j.1365-2567.2005.02276.x 16423053 PMC1782221

[45] Wang A Neill SG Newman S Tryfonidou MA Ioachimescu A Rossi MR. The genomic profiling and MAMLD1 expression in human and canines with Cushing's disease. BMC Endocr Disord. (2021) 21:185. 10.1186/s12902-021-00845-z 34517852 PMC8438999

[46] Daminet S. Canine Hypothyroidism: Update on Diagnosis and Treatment. Geneva, Switzerland: The World Small Animal Veterinary Association World Congress (2010).

[47] Spitler KM Davies BSJ. Aging and plasma triglyceride metabolism. J Lipid Res. (2020) 61:1161–7. 10.1194/jlr.R120000922 32586846 PMC7397742

[48] Subramanian S Chait A. Hypertriglyceridemia secondary to obesity and diabetes. Biochim Biophys Acta. (2012) 1821:819–25. 10.1016/j.bbalip.2011.10.003 22005032

[49] Hoffman JM Kiklevich JV Klavins K Valencak TG Austad SN. Alterations of lipid metabolism with age and weight in companion dogs. J Gerontol A Biol Sci Med Sci. (2021) 76:400–5. 10.1093/gerona/glaa186 32750116 PMC7907484

[50] Xenoulis PG Steiner JM. Lipid metabolism and hyperlipidemia in dogs. Vet J. (2010) 183:12–21. 10.1016/j.tvjl.2008.10.011 19167915

[51] Abbate SL Brunzell JD. Pathophysiology of hyperlipidemia in diabetes mellitus. J Cardiovasc Pharmacol. (1990) 16:S1–7. 10.1097/00005344-199000169-00002 1710739

[52] Teixeira FA Machado DP Jeremias JT Queiroz MR Pontieri CFF Brunetto MA. Starch sources influence lipidaemia of diabetic dogs. BMC Vet Res. (2020) 16:2. 10.1186/s12917-019-2224-y 31900155 PMC6942337

[53] Arnaldi G Scandali VM Trementino L Cardinaletti M Appolloni G Boscaro M. Pathophysiology of dyslipidemia in Cushing's syndrome. Neuroendocrinology. (2010) 92:86–90. 10.1159/000314213 20829625

[54] Sieber-Ruckstuhl NS Burla B Spoerel S Schmid F Venzin C Cazenave-Gassiot A. Changes in the canine plasma lipidome after short- and long-term excess glucocorticoid exposure. Sci Rep. (2019) 9:6015. 10.1038/s41598-019-42190-1 30979907 PMC6461633

[55] Sieber-Ruckstuhl NS Tham WK Baumgartner F Selva JJ Wenk MR Burla B. Serum lipidome signatures of dogs with different endocrinopathies associated with hyperlipidemia. Metabolites. (2022) 12:306. 10.3390/metabo12040306 35448493 PMC9031822

[56] Verkest KR. Is the metabolic syndrome a useful clinical concept in dogs? A review of the evidence. Vet J. (2014) 199:24–30. 10.1016/j.tvjl.2013.09.057 24246648

[57] Hess RS Kass PH Van Winkle TJ. Association between diabetes mellitus, hypothyroidism or hyperadrenocorticism, and atherosclerosis in dogs. J Vet Intern Med. (2003) 17:489–94. 10.1111/j.1939-1676.2003.tb02469.x 12892299

[58] Cappola AR Ladenson PW. Hypothyroidism and atherosclerosis. J Clin Endocrinol Metab. (2003) 88:2438–44. 10.1210/jc.2003-030398 12788839

[59] Poznyak A Grechko AV Poggio P Myasoedova VA Alfieri V Orekhov AN. The diabetes mellitus-atherosclerosis connection: the role of lipid and glucose metabolism and chronic inflammation. Int J Mol Sci. (2020) 21:1835. 10.3390/ijms21051835 32155866 PMC7084712

[60] Butovich IA Borowiak AM Eule JC. Comparative HPLC-MS analysis of canine and human meibomian lipidomes: many similarities, a few differences. Sci Rep. (2011) 1:24. 10.1038/srep00024 22355543 PMC3216511

[61] Kosinska MK Mastbergen SC Liebisch G Wilhelm J Dettmeyer RB Ishaque B. Comparative lipidomic analysis of synovial fluid in human and canine osteoarthritis. Osteoarthritis Cartilage. (2016) 24:1470–8. 10.1016/j.joca.2016.03.017 27049029

[62] Lloyd AJ Beckmann M Wilson T Tailliart K Allaway D Draper J. Ultra high performance liquid chromatography-high resolution mass spectrometry plasma lipidomics can distinguish between canine breeds despite uncontrolled environmental variability and non-standardized diets. Metabolomics. (2017) 13:15. 10.1007/s11306-016-1152-0 28111530 PMC5216087

[63] Corado CR Pinkstaff J Jiang X Galban EM Fisher SJ Scholler O. Cerebrospinal fluid and serum glycosphingolipid biomarkers in canine globoid cell leukodystrophy (Krabbe Disease). Mol Cell Neurosci. (2020) 102:103451. 10.1016/j.mcn.2019.103451 31794880 PMC7032565

[64] Franco J Rajwa B Gomes P HogenEsch H. Local and systemic changes in lipid profile as potential biomarkers for canine atopic dermatitis. Metabolites. (2021) 11:670. 10.3390/metabo11100670 34677385 PMC8541266

[65] Crisi PE Luciani A Di Tommaso M Prasinou P De Santis F Chatgilialoglu C. The fatty acid-based erythrocyte membrane lipidome in dogs with chronic enteropathy. Animals (Basel). (2021) 11:2604. 10.3390/ani11092604 34573570 PMC8469057

[66] Boretti FS Burla B Deuel J Gao L Wenk MR Liesegang A. Serum lipidome analysis of healthy beagle dogs receiving different diets. Metabolomics. (2019) 16:1. 10.1007/s11306-019-1621-3 31797205 PMC6890591

[67] Kalenyak K Heilmann RM van de Lest CHA Brouwers JF Burgener IA. Comparison of the systemic phospholipid profile in dogs diagnosed with idiopathic inflammatory bowel disease or food-responsive diarrhea before and after treatment. PLoS ONE. (2019) 14:e0215435. 10.1371/journal.pone.0215435 30990833 PMC6467395

[68] Hall JA MacLeay J Yerramilli M Obare E Yerramilli M Schiefelbein H. Positive impact of nutritional interventions on serum symmetric dimethylarginine and creatinine concentrations in client-owned geriatric dogs. PLoS ONE. (2016) 11:e0153653. 10.1371/journal.pone.0153653 27088214 PMC4835100

[69] Hall JA Picton RA Skinner MM Jewell DE Wander RC. The (n-3) fatty acid dose, independent of the (n-6) to (n-3) fatty acid ratio, affects the plasma fatty acid profile of normal dogs. J Nutr. (2006) 136:2338–44. 10.1093/jn/136.9.2338 16920851

[70] National Research Council Committee update of the guide for the care and use of laboratory animals. The National Academies Collection: Reports funded by National Institutes of Health. Washington (DC): National Academies Press (US), National Academy of Sciences (2011).

[71] Pan Y Larson B Araujo JA Lau W de Rivera C Santana R. Dietary supplementation with medium-chain TAG has long-lasting cognition-enhancing effects in aged dogs. Br J Nutr. (2010) 103:1746–54. 10.1017/S0007114510000097 20141643

[72] Law TH Volk HA Pan Y Zanghi B Want EJ. Metabolic perturbations associated with the consumption of a ketogenic medium-chain TAG diet in dogs with idiopathic epilepsy. Br J Nutr. (2018) 120:484–90. 10.1017/S0007114518001617 30001753 PMC6137430

[73] Hielm-Björkman A Roine J Elo K Lappalainen A Junnila J Laitinen-Vapaavuori O. An un-commissioned randomized, placebo-controlled double-blind study to test the effect of deep sea fish oil as a pain reliever for dogs suffering from canine OA. BMC Vet Res. (2012) 8:157. 10.1186/1746-6148-8-157 22950577 PMC3514349

[74] Goffin K van Maris M Corbee RJ. Effects of matrix on plasma levels of EPA and DHA in dogs. J Nutr Sci. (2017) 6:e37. 10.1017/jns.2017.30 29152241 PMC5672307

[75] Floerchinger AM Jackson MI Jewell DE MacLeay JM Paetau-Robinson I Hahn KA. Effect of feeding a weight loss food beyond a caloric restriction period on body composition and resistance to weight gain in dogs. J Am Vet Med Assoc. (2015) 247:375–84. 10.2460/javma.247.4.375 26225609

[76] Jackson MI Jewell DE. Balance of saccharolysis and proteolysis underpins improvements in stool quality induced by adding a fiber bundle containing bound polyphenols to either hydrolyzed meat or grain-rich foods. Gut Microbes. (2019) 10:298–320. 10.1080/19490976.2018.1526580 30376392 PMC6546335

[77] Quackenbush J. Microarray data normalization and transformation. Nat Genet. (2002) 32:496–501. 10.1038/ng1032 12454644

[78] Chong J Soufan O Li C Caraus I Li S Bourque G. MetaboAnalyst 40: towards more transparent and integrative metabolomics analysis. Nucleic Acids Res. (2018) 46:W486–w94. 10.1093/nar/gky310 29762782 PMC6030889

[79] R Core Team. R: A language environment for statistical computing. Vienna, Austria: R Foundation for Statistical Computing (2018). Available online at: http://www.R-project.org/ (accessed January 20, 2019).

[80] Storey JD Bass AJ Dabney A Robinson D Warnes G. qvalue: Q-value estimation for false discovery rate control. (2019). Available online at: https://github.com/StoreyLab/qvalue (accessed March 18, 2019).

[81] Bates MW. Turnover rates of fatty acids of plasma triglyceride, cholesterol ester and phospholipid in the postabsorptive dog. Am J Physiol. (1958) 194:446–52. 10.1152/ajplegacy.1958.194.3.446 13571405

[82] Watson AD Church DB Fairburn AJ. Postprandial changes in plasma urea and creatinine concentrations in dogs. Am J Vet Res. (1981) 42:1878–80. 7337284

[83] Briens JM Subramaniam M Kilgour A Loewen ME Desai KM Adolphe JL. Glycemic, insulinemic and methylglyoxal postprandial responses to starches alone or in whole diets in dogs versus cats: Relating the concept of glycemic index to metabolic responses and gene expression. Comp Biochem Physiol A Mol Integr Physiol. (2021) 257:110973. 10.1016/j.cbpa.2021.110973 33933629

[84] de Albuquerque P De Marco V Vendramini THA Amaral AR Catanozi S Santana KG. Supplementation of omega-3 and dietary factors can influence the cholesterolemia and triglyceridemia in hyperlipidemic Schnauzer dogs: A preliminary report. PLoS ONE. (2021) 16:e0258058. 10.1371/journal.pone.0258058 34665804 PMC8525743

[85] Qin Y Zhou Y Chen SH Zhao XL Ran L Zeng XL. Fish oil supplements lower serum lipids and glucose in correlation with a reduction in plasma fibroblast growth factor 21 and prostaglandin E2 in nonalcoholic fatty liver disease associated with hyperlipidemia: a randomized clinical trial. PLoS ONE. (2015) 10:e0133496. 10.1371/journal.pone.0133496 26226139 PMC4520650

[86] Siri-Tarino PW Sun Q Hu FB Krauss RM. Saturated fatty acids and risk of coronary heart disease: modulation by replacement nutrients. Curr Atheroscler Rep. (2010) 12:384–90. 10.1007/s11883-010-0131-6 20711693 PMC2943062

[87] Dias CB Garg R Wood LG Garg ML. Saturated fat consumption may not be the main cause of increased blood lipid levels. Med Hypotheses. (2014) 82:187–95. 10.1016/j.mehy.2013.11.036 24365276

[88] Pellegrino FJ Risso A Corrada Y Gambaro RC Seoane AI. Influence of dietary fish oil supplementation on DNA damage in peripheral blood lymphocytes of nine healthy dogs. Vet Rec Open. (2021) 8:e12. 10.1002/vro2.12 34188940 PMC8219285

[89] Ravić B Debeljak-Martacić J Pokimica B Vidović N Ranković S Glibetić M. The effect of fish oil-based foods on lipid and oxidative status parameters in police dogs. Biomolecules. (2022) 12:1092. 10.3390/biom12081092 36008986 PMC9405924

[90] Allaire J Couture P Leclerc M Charest A Marin J Lépine MC. A randomized, crossover, head-to-head comparison of eicosapentaenoic acid and docosahexaenoic acid supplementation to reduce inflammation markers in men and women: the Comparing EPA to DHA (ComparED) Study. Am J Clin Nutr. (2016) 104:280–7. 10.3945/ajcn.116.131896 27281302

[91] Xenoulis PG Suchodolski JS Levinski MD Steiner JM. Investigation of hypertriglyceridemia in healthy Miniature Schnauzers. J Vet Intern Med. (2007) 21:1224–30. 10.1111/j.1939-1676.2007.tb01942.x 18196730

[92] Simoens CM Deckelbaum RJ Massaut JJ Carpentier YA. Inclusion of 10% fish oil in mixed medium-chain triacylglycerol-long-chain triacylglycerol emulsions increases plasma triacylglycerol clearance and induces rapid eicosapentaenoic acid (20:5n-3) incorporation into blood cell phospholipids. Am J Clin Nutr. (2008) 88:282–8. 10.1093/ajcn/88.2.282 18689362

[93] Rouis M Dugi KA Previato L Patterson AP Brunzell JD Brewer HB. Therapeutic response to medium-chain triglycerides and omega-3 fatty acids in a patient with the familial chylomicronemia syndrome. Arterioscler Thromb Vasc Biol. (1997) 17:1400–6. 10.1161/01.ATV.17.7.1400 9261273

[94] You YQ Ling PR Qu JZ Bistrian BR. Effects of medium-chain triglycerides, long-chain triglycerides, or 2-monododecanoin on fatty acid composition in the portal vein, intestinal lymph, and systemic circulation in rats. JPEN J Parenter Enteral Nutr. (2008) 32:169–75. 10.1177/0148607108314758 18407910 PMC3202979

[95] Bibus D Lands B. Balancing proportions of competing omega-3 and omega-6 highly unsaturated fatty acids (HUFA) in tissue lipids. Prostaglandins Leukot Essent Fatty Acids. (2015) 99:19–23. 10.1016/j.plefa.2015.04.005 26002802

[96] Campbell KL Dorn GP. Effects of oral sunflower oil and olive oil on serum and cutaneous fatty acid concentrations in dogs. Res Vet Sci. (1992) 53:172–8. 10.1016/0034-5288(92)90106-C 1439206

[97] Donnelly KL Smith CI Schwarzenberg SJ Jessurun J Boldt MD Parks EJ. Sources of fatty acids stored in liver and secreted via lipoproteins in patients with nonalcoholic fatty liver disease. J Clin Invest. (2005) 115:1343–51. 10.1172/JCI23621 15864352 PMC1087172

[98] Enooku K Nakagawa H Fujiwara N Kondo M Minami T Hoshida Y. Altered serum acylcarnitine profile is associated with the status of nonalcoholic fatty liver disease (NAFLD) and NAFLD-related hepatocellular carcinoma. Sci Rep. (2019) 9:10663. 10.1038/s41598-019-47216-2 31337855 PMC6650415

[99] Bharathi SS Zhang Y Gong Z Muzumdar R Goetzman ES. Role of mitochondrial acyl-CoA dehydrogenases in the metabolism of dicarboxylic fatty acids. Biochem Biophys Res Commun. (2020) 527:162–6. 10.1016/j.bbrc.2020.04.105 32446361 PMC7248122

[100] Gregersen N Mortensen PB Kolvraa S. On the biologic origin of C6-C10-dicarboxylic and C6-C10-omega-1-hydroxy monocarboxylic acids in human and rat with acyl-CoA dehydrogenation deficiencies: in vitro studies on the omega- and omega-1-oxidation of medium-chain (C6-C12) fatty acids in human and rat liver. Pediatr Res. (1983) 17:828–34. 10.1203/00006450-198310000-00013 6634246

[101] Tserng KY Griffin RL Kerr DS. Distinction of dicarboxylic aciduria due to medium-chain triglyceride feeding from that due to abnormal fatty acid oxidation and fasting in children. Metabolism. (1996) 45:162–7. 10.1016/S0026-0495(96)90047-5 8596483

[102] Bentejac M Bugaut M Delachambre MC Lecerf J. Time-course of utilization of [stearic or lignoceric acid] sphingomyelin from high-density lipoprotein by rat tissues. Biochim Biophys Acta. (1990) 1043:134–42. 10.1016/0005-2760(90)90286-7 2317523

[103] Lisowska-Myjak B. Uremic toxins and their effects on multiple organ systems. Nephron Clin Pract. (2014) 128:303–11. 10.1159/000369817 25531673

[104] Diether NE Willing BP. Microbial fermentation of dietary protein: an important factor in diet-microbe-host interaction. Microorganisms. (2019) 7:19. 10.3390/microorganisms7010019 30642098 PMC6352118

[105] Yang CY Tarng DC. Diet, gut microbiome and indoxyl sulphate in chronic kidney disease patients. Nephrology (Carlton). (2018) 23:16–20. 10.1111/nep.13452 30298666

[106] Ephraim E Brockman JA Jewell DE. A diet supplemented with polyphenols, prebiotics and omega-3 fatty acids modulates the intestinal microbiota and improves the profile of metabolites linked with anxiety in dogs. Biology. (2022) 11:976. 10.3390/biology11070976 36101356 PMC9312346

[107] Maceyka M Spiegel S. Sphingolipid metabolites in inflammatory disease. Nature. (2014) 510:58–67. 10.1038/nature13475 24899305 PMC4320971

[108] Aichler M Borgmann D Krumsiek J Buck A MacDonald PE Fox JEM. N-acyl taurines and acylcarnitines cause an imbalance in insulin synthesis and secretion provoking beta cell dysfunction in type 2 diabetes. Cell Metab. (2017) 25:1334–47.e4. 10.1016/j.cmet.2017.04.012 28591636

