# Supplementary material for: Feeding of fish oil and medium-chain triglycerides to canines impacts circulating structural and energetic lipids, endocannabinoids, and non-lipid metabolite profiles
Source: Front Vet Sci. 2023 Aug 24;10:1168703. doi: 10.3389/fvets.2023.1168703 (PMC10484482; doi:10.3389/fvets.2023.1168703)
Supplement: Supplementary file 1 [file Table_1.DOCX]

Supplementary Material

Feeding of fish oil and medium-chain triglycerides to canines impacts circulating structural and energetic lipids, endocannabinoids, and non- lipid metabolite profiles

**Matthew I. Jackson* and Dennis E. Jewell**

*** Correspondence:** Matthew I. Jackson: [matthew_jackson@hillspet.com](mailto:matthew_jackson@hillspet.com)

**Supplementary Table 1. As-fed composition of the four food types used in the study.** Bold font indicates differences among the foods.

|  | **Food type** | | | |
| --- | --- | --- | --- | --- |
| **Ingredient, %** | **CON** | **MCT** | **FO** | **FO+MCT** |
| Fresh ground chicken | 14 | 14 | 14 | 14 |
| Low ash poultry byproduct meal | 14 | 14 | 14 | 14 |
| Pork fat | 13.9 | 6.9 | 11 | 4 |
| Pearled barley | 13.8 | 13.8 | 13.8 | 13.8 |
| Corn gluten meal | 9.6 | 9.6 | 9.6 | 9.6 |
| Whole corn | 8 | 8 | 8 | 8 |
| Whole wheat | 5.1 | 5.1 | 5.1 | 5.1 |
| Chicken liver | 5 | 5 | 5 | 5 |
| Whole sorghum | 5 | 5 | 5 | 5 |
| Palatant | 4.1 | 4.1 | 4.1 | 4.1 |
| Beet pulp fiber | 2.5 | 2.5 | 2.5 | 2.5 |
| Lactic acid | 1.2 | 1.2 | 1.2 | 1.2 |
| Soybean oil | 1 | 1 | 1 | 1 |
| Whole flax seed | 1 | 1 | 1 | 1 |
| Potassium chloride | 0.5 | 0.5 | 0.5 | 0.5 |
| Sodium chloride, iodized | 0.4 | 0.4 | 0.4 | 0.4 |
| Calcium carbonate | 0.3 | 0.3 | 0.3 | 0.3 |
| Choline chloride, 70% | 0.2 | 0.2 | 0.2 | 0.2 |
| Vitamin E, 30% | 0.2 | 0.2 | 0.2 | 0.2 |
| Vitamin mix | 0.2 | 0.2 | 0.2 | 0.2 |
| Oat fiber, fruit, vegetable blend | 0.04 | 0.04 | 0.04 | 0.04 |
| Taurine | 0.03 | 0.03 | 0.03 | 0.03 |
| **Fish oil** | **0** | **0** | **2.9** | **2.9** |
| **Medium-chain triglycerides** | **0** | **7** | **0** | **7** |

CON, control; FO, fish oil; MCT, medium-chain triglycerides.
