# Supplementary material for: Feeding of fish oil and medium-chain triglycerides to canines impacts circulating structural and energetic lipids, endocannabinoids, and non-lipid metabolite profiles
Source: Front Vet Sci. 2023 Aug 24;10:1168703. doi: 10.3389/fvets.2023.1168703 (PMC10484482; doi:10.3389/fvets.2023.1168703)
Supplement: Supplementary file 2 [file Table_2.DOCX]

Supplementary Material

Feeding of fish oil and medium-chain triglycerides to canines impacts circulating structural and energetic lipids, endocannabinoids, and non- lipid metabolite profiles

**Matthew I. Jackson* and Dennis E. Jewell**

*** Correspondence:** Matthew I. Jackson: [matthew_jackson@hillspet.com](mailto:matthew_jackson@hillspet.com)

**Supplementary Table 2. Calculated composition of food used in the study.**

|  | **Food type** | | | |
| --- | --- | --- | --- | --- |
| **Nutrient, %^a^** | **CON** | **MCT** | **FO** | **FO+MCT** |
| Dry matter | 100 | 100 | 100 | 100 |
| NFE | 34.9 | 34.8 | 34.9 | 34.8 |
| Atwater energy, kcal/kg | 4609 | 4610 | 4609 | 4610 |
| EPA+DHA | 0.02 | 0.02 | 1.5 | 1.5 |
| Calcium:phosphorus ratio | 1.1 | 1.1 | 1.1 | 1.1 |
| Arachidonic acid:EPA+DHA ratio | 8.8 | 8.4 | 0.1 | 0.1 |
| Caprylic acid (C8:0) | 0.0 | 3.7 | 0.0 | 3.7 |
| Capric acid (C10:0) | 0.02 | 4.3 | 0.01 | 4.3 |
| Arachidonic acid (C20:4n6) | 0.2 | 0.2 | 0.2 | 0.1 |
| EPA (C20:5n3) | 0.0 | 0.0 | 0.2 | 0.2 |
| DHA (C22:6n3) | 0.02 | 0.01 | 1.3 | 1.3 |
| Protein | 30.5 | 30.5 | 30.5 | 30.5 |
| Fat | 27.3 | 27.3 | 27.3 | 27.4 |
| Crude fiber | 1.6 | 1.6 | 1.6 | 1.6 |
| Ash | 5.8 | 5.8 | 5.8 | 5.8 |
| Dietary fiber | 6.8 | 6.8 | 6.8 | 6.8 |
| Soluble fiber | 1.9 | 1.9 | 1.9 | 1.9 |
| Insoluble fiber | 4.8 | 4.8 | 4.8 | 4.8 |

***^a^*** All on a dry matter basis. Data are reported as percentages except where indicated otherwise.

Caproate (C6:0) was not reported but is projected at about 8%.

CON, control; DHA, docosahexaenoate; EPA, eicosapentaenoate; FO, fish oil; MCT, medium-chain fatty acid-containing triglycerides; NFE, nitrogen-free extract.
