# Supplementary material for: Feeding of fish oil and medium-chain triglycerides to canines impacts circulating structural and energetic lipids, endocannabinoids, and non-lipid metabolite profiles
Source: Front Vet Sci. 2023 Aug 24;10:1168703. doi: 10.3389/fvets.2023.1168703 (PMC10484482; doi:10.3389/fvets.2023.1168703)
Supplement: Supplementary file 3 [file Table_3.docx]

Supplementary Material

Feeding of fish oil and medium-chain triglycerides to canines impacts circulating structural and energetic lipids, endocannabinoids, and non- lipid metabolite profiles

**Matthew I. Jackson* and Dennis E. Jewell**

*** Correspondence:** Matthew I. Jackson: [matthew_jackson@hillspet.com](mailto:matthew_jackson@hillspet.com)

**Supplementary Table 3. Characteristics of dogs in the study.**

| **Subject** | **Breed** | **Food Type** | **Sex** | **Age, y** | **Weight, kg** |
| --- | --- | --- | --- | --- | --- |
| 1 | Beagle | FO+MCT | Neutered Male | 4.0 | 11.7 |
| 2 | Beagle | FO | Spayed Female | 3.5 | 9.7 |
| 3 | Beagle | FO | Male | 3.6 | 16.3 |
| 4 | Mixed Breed | FO | Male | 1.3 | 24.4 |
| 5 | Beagle | CON | Spayed Female | 10.6 | 12.6 |
| 6 | Beagle | FO+MCT | Male | 1.9 | 11.1 |
| 7 | Beagle | CON | Neutered Male | 8.6 | 10.2 |
| 8 | Beagle | MCT | Neutered Male | 8.3 | 13.2 |
| 9 | Beagle | FO | Male | 1.0 | 12.2 |
| 10 | Beagle | CON | Spayed Female | 10.5 | 10 |
| 11 | Mixed Breed | FO+MCT | Female | 1.3 | 16.2 |
| 12 | Beagle | MCT | Male | 3.1 | 13.8 |
| 13 | Beagle | FO+MCT | Neutered Male | 10.2 | 11.3 |
| 14 | Beagle | CON | Neutered Male | 10.6 | 13.2 |
| 15 | Beagle | FO+MCT | Neutered Male | 10.4 | 10.1 |
| 16 | Beagle | MCT | Neutered Male | 3.5 | 9.2 |
| 17 | Beagle | MCT | Spayed Female | 10.6 | 8 |
| 18 | Beagle | FO | Neutered Male | 8.6 | 12.3 |
| 19 | Beagle | CON | Female | 1.0 | 9.6 |
| 20 | Beagle | FO+MCT | Spayed Female | 12.0 | 8.5 |
| 21 | Mixed Breed | MCT | Female | 1.3 | 20 |
| 22 | Beagle | FO+MCT | Male | 1.5 | 10.3 |
| 23 | Beagle | MCT | Spayed Female | 12.0 | 9.5 |
| 24 | Beagle | FO | Male | 1.2 | 16.6 |
| 25 | Beagle | CON | Neutered Male | 8.6 | 11.1 |
| 26 | Beagle | MCT | Spayed Female | 1.8 | 10.5 |
| 27 | Beagle | CON | Female | 1.2 | 11.6 |
| 28 | Beagle | FO | Spayed Female | 1.8 | 10.5 |
| 29 | Beagle | MCT | Male | 1.3 | 12.7 |
| 30 | Beagle | FO | Female | 1.2 | 8.4 |
| 31 | Beagle | MCT | Spayed Female | 5.4 | 10 |
| 32 | Beagle | FO | Spayed Female | 10.6 | 10.5 |
| 33 | Beagle | FO+MCT | Spayed Female | 2.2 | 9.6 |
| 34 | Beagle | FO+MCT | Spayed Female | 3.1 | 11.3 |
| 35 | Beagle | CON | Spayed Female | 3.6 | 9.3 |
| 36 | Beagle | FO+MCT | Spayed Female | 10.6 | 9.4 |
| 37 | Beagle | MCT | Female | 1.2 | 10.4 |
| 38 | Beagle | FO+MCT | Neutered Male | 7.0 | 9.3 |
| 39 | Beagle | CON | Spayed Female | 4.0 | 11.5 |
| 40 | Beagle | FO+MCT | Spayed Female | 10.6 | 11 |
| 41 | Beagle | FO | Spayed Female | 10.6 | 10 |
| 42 | Beagle | FO | Neutered Male | 3.1 | 9.2 |
| 43 | Beagle | CON | Male | 1.2 | 11.7 |
| 44 | Beagle | CON | Neutered Male | 4.9 | 14.6 |
| 45 | Beagle | MCT | Spayed Female | 10.1 | 11.8 |
| 46 | Beagle | MCT | Spayed Female | 10.6 | 9.7 |
| 47 | Beagle | MCT | Neutered Male | 10.2 | 14.8 |
| 48 | Beagle | FO | Neutered Male | 9.3 | 12.3 |
| 49 | Beagle | CON | Spayed Female | 10.5 | 8.8 |
| 50 | Beagle | FO | Spayed Female | 4.0 | 11.8 |
| 51 | Beagle | CON | Spayed Female | 9.3 | 8 |
| 52 | Beagle | FO+MCT | Female | 1.3 | 12.4 |
| 53 | Beagle | FO | Neutered Male | 10.6 | 10.3 |
| 54 | Beagle | FO | Spayed Female | 10.6 | 10.1 |
| 55 | Beagle | FO+MCT | Male | 1.2 | 9.3 |
| 56 | Beagle | MCT | Female | 1.3 | 12.1 |
| 57 | Beagle | FO+MCT | Neutered Male | 10.6 | 13.5 |
| 58 | Mixed Breed | CON | Male | 1.3 | 23 |
| 59 | Beagle | CON | Neutered Male | 3.1 | 10.7 |
| 60 | Beagle | MCT | Neutered Male | 10.6 | 11.7 |
| 61 | Beagle | FO+MCT | Spayed Female | 10.1 | 10.1 |
| 62 | Beagle | FO | Spayed Female | 10.2 | 10.6 |
| 63 | Beagle | MCT | Spayed Female | 3.5 | 12.9 |
| 64 | Beagle | CON | Neutered Male | 2.9 | 12.4 |

CON, control; FO, fish oil; MCT, medium-chain triglycerides.
