# Supplementary material for: Feeding of fish oil and medium-chain triglycerides to canines impacts circulating structural and energetic lipids, endocannabinoids, and non-lipid metabolite profiles
Source: Front Vet Sci. 2023 Aug 24;10:1168703. doi: 10.3389/fvets.2023.1168703 (PMC10484482; doi:10.3389/fvets.2023.1168703)
Supplement: Supplementary file 5 [file Image_1.pdf]

Supplementary Material

Article Title

Feeding of fish oil and medium-chain triglycerides to canines impacts circulating structural and energetic lipids, endocannabinoids, and non-lipid metabolite profiles

Matthew I. Jackson* and Dennis E. Jewell

*** Correspondence:** Matthew I. Jackson: matthew_jackson@hillspet.com

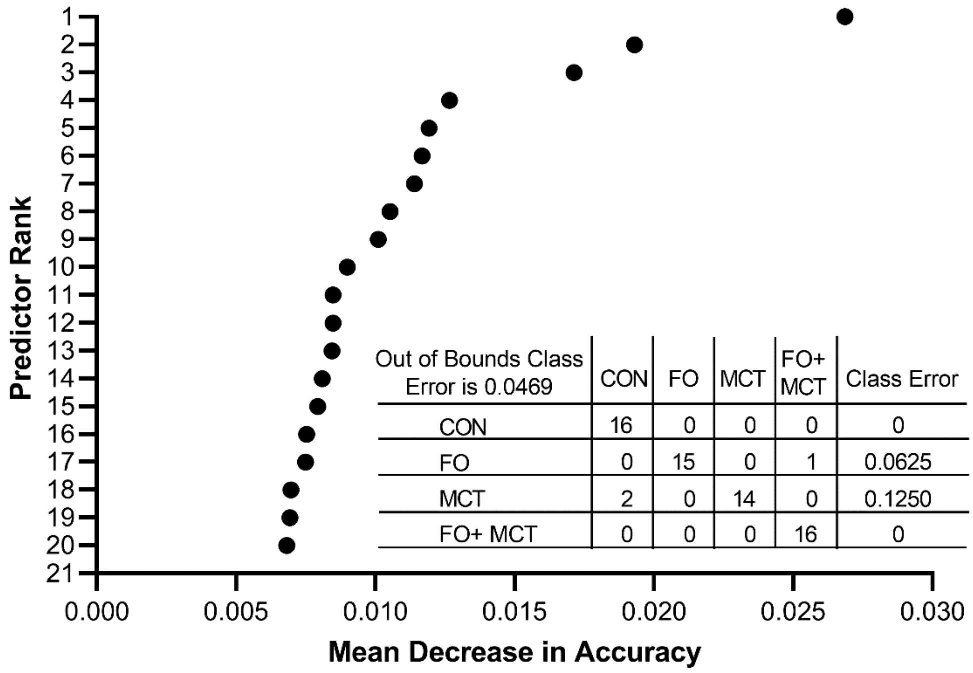

Out of Bounds Class Error is 0.0469	CON	FO	MCT	FO+ MCT	Class Error
CON	16	0	0	0	0
FO	0	15	0	1	0.0625
MCT	2	0	14	0	0.1250
FO+ MCT	0	0	0	16	0

**Metabolite**	**Predictor rank**	**Mean decrease in accuracy**
caprate 100	1	0.0269
2-hydroxylignocerate	2	0.0193
diglycerol	3	0.0171
1-palmitoyl-2-linoleoyl-GPC 160/182	4	0.0127
1-stearoyl-2-docosahexaenoyl-GPE 180/226	5	0.0119
1-oleoyl-2-docosahexaenoyl-GPC 181/226	6	0.0117
docosahexaenoate DHA 226n3	7	0.0114
1-1-enyl-palmitoyl-2-linoleoyl-GPC P-160/182	8	0.0105
1-palmitoyl-2-arachidonoyl-GPE 160/204	9	0.0101
sphingomyelin d182/231	10	0.0090
eicosapentaenoate EPA 205n3	11	0.0085
1-stearoyl-2-docosahexaenoyl-GPC 180/226	12	0.0085
heneicosapentaenoate 215n3	13	0.0084
sphingomyelin d182/241, d181/242	14	0.0081
1-palmitoyl-2-docosahexaenoyl-GPC 160/226	15	0.0079
sphingomyelin d182/242	16	0.0075
1-1-enyl-palmitoyl-2-arachidonoyl-GPC P-160/204	17	0.0075
oleoyl-arachidonoyl-glycerol 181/204 2	18	0.0070
sphingomyelin d181/202, d182/201, d161/222	19	0.0069
eicosapentaenoylcholine	20	0.0068

Supplementary Figure 1. Random Forest analysis of predictors of group differences. GPC, glycerophosphatidylcholine; GPE, glycerophosphatidylethanolamine.
